# Affordability of the EAT–*Lancet* reference diet: a global analysis

**DOI:** 10.1016/S2214-109X(19)30447-4

**Published:** 2019-11-07

**Authors:** Kalle Hirvonen, Yan Bai, Derek Headey, William A Masters

**Affiliations:** a Development Strategy and Governance Division, International Food Policy Research Institute, Bole Sub-City, Addis Ababa, Ethiopia; b Friedman School of Nutrition, Tufts University, Boston, MA, USA; c Department of Economics, Tufts University, Boston, MA, USA; d Poverty Health and Nutrition Division, International Food Policy Research Institute, Washington DC, USA

## Abstract

**Background:**

The EAT–*Lancet* Commission drew on all available nutritional and environmental evidence to construct the first global benchmark diet capable of sustaining health and protecting the planet, but it did not assess dietary affordability. We used food price and household income data to estimate affordability of EAT–*Lancet* benchmark diets, as a first step to guiding interventions to improve diets around the world.

**Methods:**

We obtained retail prices from 2011 for 744 foods in 159 countries, collected under the International Comparison Program. We used these data to identify the most affordable foods to meet EAT–*Lancet* targets. We compared total diet cost per day to each country's mean per capita household income, calculated the proportion of people for whom the most affordable EAT–*Lancet* diet exceeds total income, and also measured affordability relative to a least-cost diet that meets essential nutrient requirements.

**Findings:**

The most affordable EAT–*Lancet* diets cost a global median of US$2·84 per day (IQR 2·41–3·16) in 2011, of which the largest share was the cost of fruits and vegetables (31·2%), followed by legumes and nuts (18·7%), meat, eggs, and fish (15·2%), and dairy (13·2%). This diet costs a small fraction of average incomes in high-income countries but is not affordable for the world's poor. We estimated that the cost of an EAT–*Lancet* diet exceeded household per capita income for at least 1·58 billion people. The EAT–*Lancet* diet is also more expensive than the minimum cost of nutrient adequacy, on average, by a mean factor of 1·60 (IQR 1·41–1·78).

**Interpretation:**

Current diets differ greatly from EAT–*Lancet* targets. Improving diets is affordable in many countries but for many people would require some combination of higher income, nutritional assistance, and lower prices. Data and analysis for the cost of healthier foods are needed to inform both local interventions and systemic changes.

**Funding:**

Bill & Melinda Gates Foundation.

## Introduction

More than 2·5 billion people worldwide suffer from at least one form of malnutrition, with approximately 800 million people undernourished, around 2 billion adults overweight or obese,^1^ and over 2 billion people with micronutrient deficiencies.^2^ Poor-quality diets are now the leading cause of morbidity and mortality in the world,^3^ due to both inadequate consumption of nutritious foods and excess consumption of harmful ones. Current food production methods pose risks to the health of the planet as well. The agricultural sector now accounts for 16–27% of global greenhouse-gas emissions and is a major cause of freshwater pollution, soil degradation, and loss of biodiversity.^4^ The global food system falls far short of achieving global goals for both health and the environment.

Realigning food systems to deliver better health and environmental outcomes is therefore among the most important global challenges of the 21st century. To help guide change, the EAT–*Lancet* Commission^5^ was tasked with using the best available evidence to determine a universal reference diet that is healthy for both humans and the planet, minimising chronic disease risks and maximising human wellbeing. The EAT–*Lancet* reference diet is rich in fruits and vegetables, with protein and fats sourced mainly from plant-based foods and unsaturated oils from fish, and carbohydrates from whole grains. Combined with improved agricultural production practices and a reduction of food waste and loss, the Commission estimated that this diet would permit feeding the estimated 10 billion people in 2050 within planetary boundaries that restrict global warming, land-systems change, freshwater expansion, biodiversity loss, and nitrogen and phosphorus cycling.^5^

A shift to healthier diets requires that the necessary foods be both available and affordable for low-income populations.^6^ This factor is recognised by the EAT–*Lancet* Commission, although the report did not consider cost or affordability of the healthy reference diet. To improve dietary intake, the EAT–*Lancet* Commission calls for a Great Food Transformation: “a substantial change in the structure and function of the global food system so that it operates with different core processes and feedback”. In this study, we aimed to provide evidence to guide those changes by calculating the most affordable way to meet EAT–*Lancet* targets using available foods in almost every country of the world, and comparing the resulting dietary cost to prevailing incomes in each country.

**Research in context**

**Evidence before this study**

The EAT–*Lancet* Commission on healthy diets from sustainable food systems was published on Jan 16, 2019, providing the first evidence-based targets for a healthy and environmentally sustainable diet. The Commission did not address cost or affordability, and a PubMed search for “EAT *Lancet*” on June 13, 2019, did not show any other research into these issues.

**Added value of this study**

This is the first study, to our knowledge, to calculate the cost of foods needed for a healthy and sustainable diet across the globe. Using standardised data for prices for 744 food items in 159 countries, the minimum daily cost of an EAT–*Lancet* reference diet in 2011 (in international dollars) ranged from a median of $2·42 in low-income countries to $2·66 in high-income countries. These reference diets are affordable for most of the world's people, but not in low-income countries where the cheapest food options for meeting EAT–*Lancet* targets would cost nearly 90% of the mean per capita household income. For at least 1·58 billion people, mostly in sub-Saharan Africa and South Asia, the cost of this reference diet would exceed their total income. Reaching EAT–*Lancet* targets would cost an average of 60% more than the least-cost options for achieving adequate intake of essential nutrients. The EAT–*Lancet* reference diet is often unaffordable for the poor because it requires larger quantities of higher-cost food groups such as dairy, eggs, meat, fish, fruits, and vegetables than the near-subsistence diets that are consumed by very poor people.

**Implications of all the available evidence**

Our findings indicate that a widespread global shift to the EAT–*Lancet* diets is feasible only through some combination of higher earnings, more favourable market prices, and nutrition assistance for low-income people, in addition to changes in local and global food systems that drive food choice among more affluent populations. Meeting EAT–*Lancet* targets in low-income areas will require higher farm productivity and lower food prices, plus greater non-farm earnings and social safety nets, allowing people to shift consumption away from starchy staples and increase their intake of more nutritious but currently unaffordable animal-sourced and vegetal foods.

## Methods

### Overview

The analysis took place in three stages. In the first stage, we used detailed information on locally available foods in all countries for which data were available to identify the lowest-cost items needed to meet the EAT–*Lancet* reference diet (table 1). In the second stage, we identified for whom total cost is affordable or unaffordable based on household incomes. We compared the resulting daily cost to average daily household income per capita and used the same survey-based evidence on the distribution of income to calculate the number of people for whom the daily cost of an EAT–*Lancet* reference diet is not currently affordable. In the third stage, we compared the local cost of EAT–*Lancet* reference diets in each country to the least-cost combination of foods that meet daily requirements of 20 essential nutrients.^7^ Taken together, these results identify the specific regions and food groups in which locally available items that are needed to meet EAT–*Lancet* targets are affordable, or not affordable, to inform both local interventions and systemic changes.

**Table 1:** Composition of the EAT–*Lancet* reference diet, by food group

	**Serving, kcal per day (g)**	**Functional category**	**Serving, kcal per day**	**Broad food groups**	**Serving, kcal per day**
Rice, wheat, corn, and other	811 (232 g)	Rice, wheat, corn, and other	811	Starchy staples	850
Potatoes and cassava	39 (50 g)	Potatoes and cassava	39	..	..
Dark green vegetables	100 (23 g)	Dark green vegetables	23	Fruits and vegetables	204
Red and orange vegetables	30 (100 g)	Red and orange vegetables	30	..	..
Other vegetables	25 (100 g)	Other vegetables	25	..	..
All fruits	126 (200 g)	All fruits	126	..	..
Whole milk or equivalents	153 (250 g)	Whole milk or equivalents	153	Dairy	153
Beef and lamb	15 (7 g)	Beef, lamb, and pork	30	Meat, eggs, and fish	151
Pork	15 (7 g)	..	..	..	..
Chicken and other poultry	62 (29 g)	Poultry, eggs, and fish	121	..	..
Eggs	19 (13 g)	..	..	..	..
Fish	40 (28 g)	..	..	..	..
Dry beans, lentils, and peas	172 (50 g)	Legumes, nuts, and soy foods	575	Legumes and nuts	575
Soy foods	112 (25 g)	..	..	..	..
Peanuts	142 (25 g)	..	..	..	..
Tree nuts	149 (25 g)	..	..	..	..
Palm oil	60 (6·8 g)	Palm oil	60	Oils and fats	450
Unsaturated oils	354 (40 g)	Unsaturated oils	354	..	..
Dairy fats	0 (0 g)	Dairy fats	0	..	..
Lard or tallow	36 (5 g)	Lard or tallow	36	..	..
All sweeteners	120 (31 g)	All sweeteners	120	Sweeteners	120
Total	2503	Total	2503	Total	2503

Data are daily servings in grams or kcal. The healthy reference diet described by Willett and colleagues^5^ is reported in the left column (EAT–*Lancet* groups). The functional category allows for exchangeability across certain EAT–*Lancet* food groups. The broad food group provides the broad aggregations of the EAT–*Lancet* food groups.

### The EAT–*Lancet* reference diet

The reference diet recommended by Willett and colleagues^5^ is described in the table 1. This EAT–*Lancet* reference diet provides 2503 kcal per day, corresponding to the average energy needs of a 30-year-old woman weighing 60 kg and whose physical activity level is between moderate and high. The specific serving sizes for each food group are derived from the best available scientific evidence about both health risks and environmental effects of different foods. Relative to current consumption in most high-income countries, these serving sizes are small for animal source foods but large for fruits and vegetables.^5^ The EAT–*Lancet* report also specifies which food groups might be substituted for each other, based on their nutritional content and environmental effect. For example, poultry is exchangeable with fish and eggs, and various plant-based protein sources are interchangeable. The resulting food groups (table 1) allow flexibility by combining red meat (beef, lamb, and pork), white meat, fish, and eggs (poultry, fish, and eggs), and legumes and nuts (dry beans, soy foods, and peanuts). However, we did not consider the food-group-specific uncertainty ranges provided by the Commission because of the ambiguity of how to substitute across food groups within these ranges.

### Food prices and availability by country

The availability and cost of acquiring goods and services around the world is monitored by the International Comparison Program (ICP), a collaboration between the World Bank and country statistical agencies charged with collecting nationally representative prices for widely consumed goods and services. The purpose of the ICP is to standardise price collection, for use in measuring economic activity, poverty rates, and purchasing power parity exchange rates between currencies.^8^ The ICP aims for global coverage and includes sufficient data for this project from 159 countries accounting for 95% of the world's population (appendix). The ICP data can be obtained for research purposes by application to the World Bank.^9^

The most recent available ICP data are from 2011, with prices for a globally standardised list of 199 foods and non-alcoholic beverages, which we supplemented with an additional 545 region-specific items for which the ICP regional authorities collated prices within Africa, Asia and the Pacific, Latin America and the Caribbean, and western Asia. The resulting list of 744 items in 159 countries yielded 21 121 price observations. Almost all items without a price observation were considered unavailable for purchase. The only exception is that 38 of the higher-income countries did not report any price for basic starchy staples such as potatoes or rice, for which we imputed their cost as the mean price from a country's geographical subregion (appendix).

We grouped all ICP items to EAT–*Lancet* food groups (appendix). We then matched each ICP item with the corresponding food item in the US Department of Agriculture National Nutrient Database^10^ to obtain the item's edible portion and energy contents (kcal). After this, we calculated the item's price per kcal considering the edible portion and computed the most affordable way to meet EAT–*Lancet* targets from locally available foods using the least expensive item within each EAT–*Lancet* food group. In rare cases where a country reported no prices for any item within one of the EAT–*Lancet* groups, we substituted the lowest cost item within the closest other food category (appendix). For some countries, the ICP data include prices for whole animals, such as fish and poultry, that are not in the US Department of Agriculture product list. In these instances, we obtained estimates of edible portions from the Food and Agriculture Organization of the United Nations and International Network of Food Data Systems (appendix).^11^

Finally, to compare costs across countries, we converted local currency prices to 2011 international dollars using purchasing power parity exchange rates for household consumption, which measure the amount of local currency units needed to purchase the same bundle of goods and services in each country. Purchasing power parity conversions are based on the same price data used to compute diet costs but are an average of all food and non-food items in proportion to their importance for the country's total spending.

### Income and affordability by country

To measure affordability, we used survey-based estimates of countries' mean daily per capita household income from the World Bank's PovcalNet system,^12^ which covers 141 countries accounting for 92% of the world's population (appendix). The PovcalNet data are based on household consumption for most low-income and middle-income countries and on household income for all high-income countries (appendix). Using surveys to ask people about their earnings is typically more feasible in higher-income countries. In low-income and middle-income countries, asking about total consumption is often preferable in part because many households obtain food in kind from their own farms.^13^ For convenience, we used the term income to mean both earnings and consumption. We also reported affordability relative to the country's gross national income, which is calculated from national accounts and includes the cost of public services as well as household income.^14^ Because survey-based estimates are the more relevant benchmark for affordability of each diet,^15^, ^16^ results using gross national income for 156 countries are provided in the appendix. Focusing on the survey data also allowed us to calculate the number of people in each country for whom the lowest-cost way to meet the EAT–*Lancet* reference diet exceeded the total value of all household income per capita (appendix).

Our second benchmark of affordability was to compare EAT–*Lancet* diets against the least-cost way to obtain adequate levels of essential nutrients, without consideration of additional attributes associated with EAT–*Lancet* food groups. This cost of nutrient adequacy is an updated version of the least-cost diet concept originally suggested by Stigler^17^ that has since been used for a wide range of purposes.^18^, ^19^, ^20^, ^21^, ^22^ To compare with the EAT–*Lancet* reference diet, we defined the cost of nutrient adequacy as the lowest-cost combination of foods needed to meet all requirements of 20 essential nutrients for a healthy 60 kg woman at 30 years old, in energy balance at 2503 kcal per day (appendix). The quantity of each food needed to deliver nutrients in the required proportions was calculated by linear programming, to give a lower bound on the daily cost of meeting a healthy woman's minimum estimated average requirements, while staying below the maximum upper level of toxicity risk for each micronutrient and within acceptable macronutrient distribution range for protein, fats, and carbohydrates.^7^ The foods selected for the cost of nutrient adequacy are not a recommended diet because it makes no provision for attributes other than essential nutrients. This nutrients-only diet serves as a useful benchmark to measure the additional cost (beyond those 20 nutrients) of meeting EAT–*Lancet* targets in each country.

### Statistical analysis

We grouped countries in the figures and tables by the size of their economy (high-income, upper-middle-income, lower-middle income, and low-income countries) and by their geographical location (east Asia and Pacific, Europe and central Asia, Latin America and the Caribbean, Middle East and North Africa, North America, south Asia, and sub-Saharan Africa) using World Bank classifications (appendix has definitions of these income groups and the list of countries in each group). We used Stata (version 15) for all statistical analyses.

### Role of the funding source

The funder had no role in the study design, data analysis, interpretation, or writing of the report. All authors had full access to all the data in the study and had final responsibility for the decision to submit for publication.

## Results

**Figure 1:**
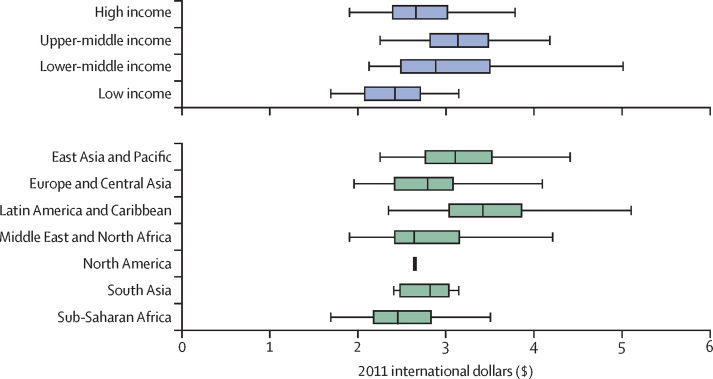
Cost of the EAT–*Lancet* reference diet in 2011 international dollars, by country income levels and major regions We used price data from the International Comparison Program to estimate the cost of the EAT–*Lancet* reference diet in 159 countries. Cost estimates are reported in 2011 international dollars, adjusting for inflation using purchasing power parity price levels for household consumption. The size of the box indicates the IQR. The bottom and top rule marks the bottom 5th and top 5th percentiles, respectively. The vertical bar rule inside the box shows the median value for the income group or geographical region.

The median daily cost in international dollars of an EAT–*Lancet* reference diet was estimated to be $2·84 (IQR 2·41–3·16) in 2011. The cost was larger in high-income countries ($2·66, 2·39–3·02) than in low-income countries ($2·42, 2·07–2·72), and among geographical regions, the median cost was highest in the Latin America and Caribbean region ($3·42, 3·03–3·87) and lowest in sub-Saharan Africa ($2·45, 2·17–2·84), with considerable variation within regions and income groups (figure 1).

The food group whose quantities and prices accounted for the largest share of total cost (31·2%) was fruits and vegetables (table 2). In high-income countries, this share was on average 35·1%, in upper-middle-income countries 30·3%, in lower-middle-income countries 29·7%, and in low-income countries 26·7%. Globally the next largest cost is from legumes and nuts (18·7%), meat, eggs, and fish (15·2%), and dairy (13·2%). Adding together all animal sourced food groups (dairy, plus meat, eggs, and fish), their share of total cost was largest in low-income countries (32·8%) and smallest in upper middle-income countries (26·2%).

**Table 2:** Cost components of the EAT–*Lancet* reference diet, by EAT–*Lancet* food groups in 2011 international dollars, by country income levels and major regions

		**Starchy staples**	**Legumes and nuts**	**Fruits and vegetables**	**Dairy**	**Meat, eggs, and fish**	**Oils and fats**	**Sweeteners**	**Total**
Serving (kcal/day)		850	575	204	153	151	450	120	2503
Global (n=159)		$0·32 (11·2%)	$0·54 (18·7%)	$0·90 (31·2%)	$0·38 (13·2%)	$0·44 (15·2%)	$0·23 (8·0%)	$0·07 (2·5%)	$2·89 (100%)
By country income level									
	High income (n=52)	$0·30 (11·0%)	$0·55 (19·7%)	$0·97 (35·1%)	$0·33 (11·8%)	$0·41 (14·7%)	$0·16 (5·8%)	$0·05 (1·9%)	$2·77 (100%)
	Upper-middle income (n=40)	$0·37 (11·7%)	$0·66 (20·7%)	$0·97 (30·3%)	$0·38 (12·0%)	$0·45 (14·2%)	$0·27 (8·6%)	$0·08 (2·4%)	$3·20 (100%)
	Lower-middle income (n=41)	$0·34 (11·3%)	$0·53 (17·6%)	$0·91 (29·7%)	$0·46 (15·0%)	$0·48 (15·6%)	$0·25 (8·2%)	$0·08 (2·6%)	$3·05 (100%)
	Low income (n=26)	$0·25 (10·1%)	$0·35 (14·6%)	$0·65 (26·7%)	$0·38 (15·4%)	$0·42 (17·4%)	$0·28 (11·7%)	$0·10 (4·2%)	$2·43 (100%)
By geographical region									
	East Asia and Pacific (n=20)	$0·36 (11·1%)	$0·54 (16·5%)	$1·17 (35·9%)	$0·58 (17·6%)	$0·38 (11·5%)	$0·18 (5·5%)	$0·07 (2·0%)	$3·27 (100%)
	Europe and central Asia (n=45)	$0·27 (9·5%)	$0·55 (19·4%)	$0·94 (32·8%)	$0·35 (12·2%)	$0·47 (16·4%)	$0·22 (7·7%)	$0·06 (2·1%)	$2·86 (100%)
	Latin America and Caribbean (n=23)	$0·46 (13·3%)	$0·90 (25·9%)	$1·02 (29·2%)	$0·34 (9·6%)	$0·46 (13·3%)	$0·22 (6·4%)	$0·08 (2·4%)	$3·48 (100%)
	Middle East and north Africa (n=17)	$0·35 (12·5%)	$0·50 (17·5%)	$0·78 (27·7%)	$0·36 (12·9%)	$0·51 (18·1%)	$0·25 (8·7%)	$0·07 (2·4%)	$2·83 (100%)
	North America (n=2)	$0·47 (17·7%)	$0·40 (15·0%)	$0·68 (25·8%)	$0·33 (12·3%)	$0·35 (13·1%)	$0·36 (13·6%)	$0·07 (2·5%)	$2·65 (100%)
	South Asia (n=7)	$0·29 (10·2%)	$0·46 (16·6%)	$0·88 (31·3%)	$0·39 (13·8%)	$0·53 (18·9%)	$0·19 (6·8%)	$0·07 (2·4%)	$2·80 (100%)
	Sub-Saharan Africa (n=45)	$0·27 (10·9%)	$0·38 (15·2%)	$0·74 (29·6%)	$0·36 (14·6%)	$0·39 (15·4%)	$0·27 (10·7%)	$0·09 (3·6%)	$2·50 (100%)

Data are mean cost of 2011 international dollars at the purchasing power parity price level for household consumption (% are of the total cost). Due to rounding, the numbers do not always add up precisely to the totals reported in the last column. The food grouping is based on the broad food groups reported in table 1.

**Figure 2:**
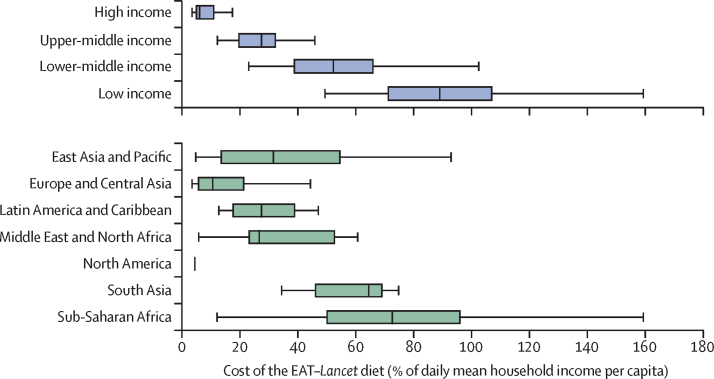
Cost of the EAT–*Lancet* reference diet relative to mean daily per capita household income by country income levels and major regions We used price data from the International Comparison Program to estimate the cost of the EAT–*Lancet* diet and compared these estimates to mean daily per capita household income. The size of the box indicates the IQR. The bottom and top rule marks the bottom fifth and top fifth percentiles, respectively. The vertical bar rule inside the box shows the median value for the income group or geographical region. N=141 countries.

The affordability of EAT–*Lancet* reference diets, as a proportion of mean daily household income per capita, was 6·1% (IQR 4·8–11·2) in high-income countries, 27·5% (19·5–32·5) in upper-middle-income countries, 52·4% (38·7–66·2) in lower-middle-income countries, and 89·1% (71·1–107·3) in low-income countries (figure 2).

Among regions, diet cost as a fraction of mean daily per capita household income was lowest in North America (4·42%; IQR 4·21–4·63) and highest in sub-Saharan Africa (72·73%; 49·92–96·28). Geographical variation was considerable even within regions (appendix), and the estimated cost of an EAT–*Lancet* reference diet exceeded the mean daily per capita household income in Burkina Faso, Burundi, Democratic Republic of the Congo, Guinea-Bissau, Lesotho, Madagascar, Malawi, Nigeria, Sierra Leone, and Yemen. The affordability estimates based on daily per capita gross national income supported the ranking of regions and income groups, although the estimates based on gross national income indicated somewhat better affordability in all countries (appendix).

Measuring affordability relative to household per capita income, we estimated that the cost of an EAT–*Lancet* reference diet exceeds total income for at least 1·58 billion people, out of which 80% (1·26 billion) are in middle-income countries (table 3). The prevalence of individuals with total household income per person below the estimated least-cost of the EAT–*Lancet* reference diet is highest in sub-Saharan Africa (57·2%) followed by south Asia (38·4%).

**Table 3:** Number and share of people with daily income below the cost of the EAT–*Lancet* reference diet, by country income levels, and major regions

		**Number of countries**	**Population (in millions)**	**Share (%)**
**Global**		**141**	**1579·02**	**23·8%**
**By country income level**				
	High income	38	9·00	0·8%
	Upper-middle income	37	254·07	10·8%
	Lower-middle income	40	1005·89	37·1%
	Low income	26	310·06	62·2%
**By geographical region**				
	East Asia and Pacific	13	319·88	15·0%
	Europe and central Asia	45	14·86	1·7%
	Latin America and Caribbean	19	62·84	11·6%
	Middle East and North Africa	11	48·40	19·4%
	North America	2	3·95	1·2%
	South Asia	7	627·31	38·4%
	Sub-Saharan Africa	44	501·77	57·2%

We used the World Bank's PovcalNet system to calculate the share of people in each country whose daily consumption or income was less than the estimated cost of the EAT–*Lancet* reference diet.

**Figure 3:**
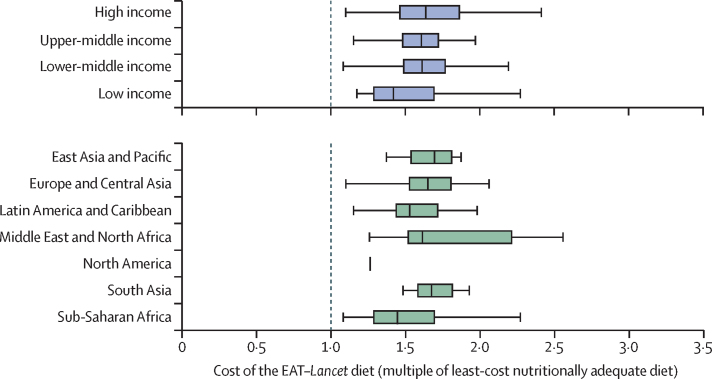
Comparing the cost of EAT–*Lancet* reference diets to the minimum cost of nutrient adequacy, by level of national income or geographical region We used price data from the International Comparison Program to estimate the cost of the EAT–*Lancet* diet in 159 countries, and computed the cost of meeting only estimated average requirements, upper limits and average macronutrient distribution ranges for essential nutrients. At the dashed vertical line, the two diets would have identical cost. Data shown are the cost of an EAT–*Lancet* diet as a multiple of the nutrients-only diet—for example, a value of 1·5 represents a 50% higher cost. The size of the box indicates the IQR. The bottom and top rule marks the bottom fifth and top fifth percentiles, respectively. The vertical bar rule inside the box shows the median value for the income group or geographical region. N=159 countries.

Measuring affordability relative to alternative sources of essential nutrients, an EAT–*Lancet* reference diet is more expensive by a factor of 1·60 (IQR 1·41–1·78). The added cost of meeting EAT–*Lancet* standards varies widely across income levels and geographical regions (figure 3). The main cost differences between the EAT–*Lancet* reference diet and the cost of nutrient adequacy largely originate from the larger quantity of animal source foods in the EAT–*Lancet* reference diet than would be required for nutrient adequacy alone (appendix).

## Discussion

Our study showed that EAT–*Lancet* reference diets are not affordable for much of the world's low-income population. In the 26 countries (0·50 billion people) classified as low-income by the World Bank, obtaining enough of the least expensive locally available items to meet EAT–*Lancet* targets would require 89·1% of the mean per capita household income. In the 47 countries (2·97 billion people) classified as lower-middle income, these diets would cost 52·4% of the mean per capita household income.

We estimated that at least 1·58 billion individuals, mostly located in sub-Saharan Africa and south Asia, face a daily cost of meeting EAT–*Lancet* targets in their country that exceeds their total per capita household income. Although large, this estimate of 1·58 billion is a lower bound because many more people would be unable to afford EAT–*Lancet* reference diets after paying for non-food necessities such as housing, transportation, education, and health care. Furthermore, we found that EAT–*Lancet* reference diets were on average 60% more costly than the foods needed for nutrient adequacy, due, in part, to larger quantities of animal-source foods as well as fruits and vegetables. The EAT–*Lancet* Commission recommends a diet containing less meat than diets currently consumed by richer people but includes more of these high-cost foods than the world's poor now consume or could afford.

Making the EAT–*Lancet* reference diet more affordable for the poor would require some combination of higher incomes and lower prices, without which individuals cannot obtain sufficient quantities from each food group. Lower prices could come from improvements in local production, marketing, and trade, and expanding the range of lower-cost options in each food group. Inclusive economic growth is needed for poor households to afford a larger quantity of more nutritious foods, but variation in prices and income ensures that targeted investments in nutritional assistance and social safety nets would also be needed for food insecurity and malnutrition to be eliminated.

Beyond affordability for the world's poor, many other changes would be needed for people to choose an EAT–*Lancet* diet. Drivers of choice among affordable items include individually modifiable factors, such as time and convenience, nutrition knowledge, and acquired tastes and habits, which in turn are shaped by societal factors such as marketing practices, as well as forces outside the food system, including child care, housing, and transportation. The Great Food Transformation described in the EAT–*Lancet* Commission rightly calls for change not only in prices and purchasing power but also in many other factors described in the vast published literature on food choice.^23^

Looking across food groups, our analysis confirms that fruits and vegetables and animal source foods are the most expensive components of the EAT–*Lancet* reference diet,^24^ and that retail markets in lower-income countries have some less expensive vegetal foods but more expensive animal source foods than are available in higher-income countries. Other analyses of ICP price data confirm this pattern,^25^, ^26^ which could be explained by differences in productivity and farm-to-retail food systems in higher-income countries that feature specialised investment to supply eggs, milk, fish, and other animal-sourced foods at lower unit cost.^27^ Increasing access to animal source foods could be helpful for children in low-income countries;^25^ our analysis focuses on adult diets, showing that access to inexpensive vegetal foods allows adults to meet essential nutrient requirements with even less animal source foods than the quantities specified in the EAT–*Lancet* reference diet.

This study has several limitations. First, for each country, we provide only a lower bound on the cost of EAT–*Lancet* reference diets, based on the most affordable item in each food group. Even low-income consumers might choose a variety of more expensive foods in each group, consuming an EAT–*Lancet* reference diet that also meets other goals, such as speed and ease of preparation as well as cultural preferences. Second, our cost and affordability estimates are designed to provide national and global totals for the most recent available year, masking spatial heterogeneity within countries as well as variation over time. Cost-of-living differences are substantial between rural and urban areas,^28^ and are further complicated by differences in availability of different items.^29^ Households with their own farms, gardens, or livestock might access their own production some of the year, and seasonality plays an important role in food prices and availability for food buyers as well.^30^ A third kind of limitation concerns variation in nutritional needs because the EAT–*Lancet* reference diet and our cost of nutrient adequacy calculations pertain only to a typical adult woman, considers a limited set of nutrients, and overlooks differences in bioavailability across food groups. Demographically disaggregated analyses considering a wider set of nutrients and accounting for bioavailability would improve the quality of these dietary affordability metrics. Finally, the nutritional content is uncertain for each item for which a price is reported, and although our list of 744 distinct foods includes many diverse foods, other foods might exist that would be less costly than those for which prices are reported to the ICP. These limitations suggest the need for more in-depth analyses of dietary costs that capture differences in the affordability of an extensive range of foods measured across locations and over time, and with individuals with different calorie requirements.

Economic extensions to the EAT–*Lancet* research agenda can offer important insights into the specific interventions and systemic changes needed to improve diets. Even if many poor consumers were to aspire to consume healthier and more environmentally sustainable foods, income and price constraints frequently render this diet unaffordable. Measures to alleviate price and income constraints will be essential to bringing healthy and sustainable diets within reach of the world's poor.
