# Correction to *Lancet Glob Health* 2020; 8: e59–66

**DOI:** 10.1016/S2214-109X(20)30472-1

**Published:** 2020-11-18

*Hirvonen K, Bai Y, Headey D, Masters WA. Affordability of the EAT–Lancet reference diet: a global analysis.* Lancet Glob Health *2020; **8:** e59–66—*In this Article, labelling of units in Table 1 column 1 for serving and kcal per day of dark green vegetables has been corrected. This correction has been made as of Nov 18, 2020.

