# Supplementary material for: Affordability of the EAT–Lancet reference diet: a global analysis
Source: Lancet Glob Health. 2019 Nov 7;8(1):e59–66. doi: 10.1016/S2214-109X(19)30447-4 (PMC7024996; doi:10.1016/S2214-109X(19)30447-4)
Supplement: Supplementary appendix [file mmc1.pdf]

THE LANCET
Global Health

Supplementary appendix

This appendix formed part of the original submission and has been peer reviewed. We post it as supplied by the authors.

Supplement to: Hirvonen K, Bai Y, Headey D, Masters WA. Affordability of the EAT–*Lancet* reference diet: a global analysis. *Lancet Glob Health* 2019; published online Nov 7. http://dx.doi.org/10.1016/S2214-109X(19)30447-4.

Affordability of healthy and sustainable diets: Global analysis of the EAT-Lancet reference diet relative to household income and the cost of nutrient adequacy

Supplemental Material

Contents:

Appendix 1: ICP food price data 2
Appendix 2: Imputation and substitution for missing ICP prices 6
Affluent countries with missing starchy staple prices 6
Food groups with few price observations 8
Appendix 3: Food composition tables 9
Appendix 4: Additional details on Povcal data 10
Appendix 5: Affordability estimates based on daily per capita gross national income (GNI) . 15
Appendix 6: Cost of Nutrient Adequacy (CoNA) 18
Appendix 7: World Bank's country classifications 19
Appendix 8: Cost of the EAT-Lancet reference diet relative to mean daily per capita household income, map 24
Appendix 9: Composition of reference diets for EAT-*Lancet* and minimum cost of nutrient adequacy 25
References 44

Appendix 1: ICP food price data

The International Comparison Program (ICP) is a global partnership of local and regional statistical offices in 199 countries, led by the World Bank to improve and standardize the collection and reporting of prices for all goods and services in the global economy. The primary purpose of these data is to construct price indexes with which to measure the real size and composition of economic activity, poverty rates and other indicators of well-being (1). Comparisons require collecting prices for the same item in multiple countries, to determine each country's overall price level and permit conversion of its currency to international dollars in purchasing power parity (PPP) terms, meaning that one dollar would buy an equal quantity of good and services in each country. ICP data collection uses a global list of specific items for which prices are sought in all countries of the world, complemented by regional lists of location-specific items. Missing data implies that an item is not typically available for purchase in a given country.

For this study we used the latest available round of ICP data, providing prices observed in 2011. Of the 199 ICP member countries, we excluded Georgia and Iran because they did not take part in ICP's regional comparisons, and excluded 38 other countries -- mostly small island countries in the Pacific and the Caribbean – because of incomplete reporting to the ICP. The final sample used in the analysis is 159 countries, accounting for 95 % of the world's population in 2017. The populations omitted from our analysis due to incomplete reporting to the ICP are listed in Table S1.

Table S1: Countries dropped from the analysis

Country	Population
American Samoa	55,640
Anguilla	14,764
Antigua and Barbuda	102,010
Aruba	105,260
Bahamas, The	395,360
Barbados	285,720
Belize	374,680
Bermuda	65,440
Bonaire	18,905
Cayman Islands	61,560
Cook Islands	17,459
Curaçao	161,010
Dominica	73,920
French Polynesia	283,010
Georgia	3,717,100
Grenada	107,830
Guam	164,230
Iran, Islamic Republic	81,162,790
Kiribati	116,400
Marshall Islands	53,130
Micronesia, Federated States of	105,540

Country	Population
Montserrat	5,215
Nauru	13,650
New Caledonia	280,460
Niue	1,624
Northern Mariana Islands	55,140
Palau	21,730
Papua New Guinea	8,251,160
Samoa	196,440
Sint Maarten	41,110
Solomon Islands	611,340
St. Lucia	178,840
St. Vincent and the Grenadines	109,900
Tokelau	1,499
Tonga	108,020
Turks and Caicos Islands	35,450
Tuvalu	11,190
Vanuatu	276,240
Virgin Islands, British	31,200
Wallis and Futuna	11,899
**Total**	**97,683,865**

Note: Population estimates are for 2017 and sourced from UN (2).

Prices in the ICP dataset are reported using the UN Statistical Agencies system of Classification of Individual Consumption According to Purpose (COICOP), and we retained prices for all items classified in COICOP as foods and beverages. Prices are observed and reported to ICP by national statistical agencies, based on the following guidelines (see 1):

1. Spatial representativeness so that all economic areas (including rural and urban) of the country are represented.
2. Temporary representativeness: prices are collected several times during the year to account for seasonality in prices.
3. Representativeness in terms of outlets, ranging from supermarkets to small kiosks.
4. Selection of products: under each basic heading[1], countries were asked to select at least one product from the global list and three most important products in the regional list.

[1] Basic headings are used to group similar (food and beverage) items.

We were able to obtain the price data covering the global product list and the regional lists for Africa, Asia and the Pacific, Latin America, the Caribbean, and Western Asia. However, for 59 countries in the CIS, Eurostat-OECD, and the Pacific Islands regions we have to rely only on the price data collected using the global product list.

To calculate quantities required for EAT-*Lancet* reference diets, we matched the ICP descriptions of each item to their food composition. Table S2 shows the total number of food items in the ICP's global and regional lists. Out of the 823 items reported to the ICP in the COICOP categories for foods and beverages, we were able to match 744 (90.4 %) with their calorie content in the USDA database (see appendix 3).

Table S2: Number of ICP price observations in our combined global and regional database

	Number of observations		Matched to calorie estimates in USDA
	Countries	Foods	
Global list		201	199
Africa only	50	203	175
Asia only	23	167	144
West Asia only	12	177	162
LAC only	16	75	64
**Combined (global and regional)**	**180**	**823**	**744**

We then categorized the 744 ICP items to the EAT-*Lancet* food groups, see table S3 at the end of this supplement. From the full ICP food and beverage list, 548 items were categorized into the 15 functional food groups (see table 1 in the manuscript). There were 196 items that were not matched with the EAT food groups and are therefore not considered in the analysis, primarily spices, condiments, and confectionery products such as candy.

Table S4 summarizes the number of ICP price observations in each food group. In total, we have 21,121 price observations across 159 countries. We see that the ICP has an extensive coverage of certain food groups such as cereals, legumes, fruits, vegetables and animal source foods with more than 1,000 observations in each food group. In contrast, dark green vegetables, palm oil, and animal fats (lard or tallow) have relatively fewer price observations.

Table S4: Number of price observations by EAT-*Lancet* food group

Food group	Serving size (kcal/day)	N	%
Rice, wheat, corn & other cereals	811	4,198	19.88
Potatoes and cassava	39	402	1.9
Dark green vegetables	23	350	1.66
Red & orange vegetables	30	629	2.98
Other vegetables	25	1,620	7.67
All fruits	126	2,398	11.35

Whole milk or derivatives	153	1,928	9.13
Beef, lamb & pork	30	2,990	14.16
Poultry, eggs & fish	121	3,826	18.11
Legumes, nuts & soy foods	575	1,057	5
Palm oil	60	97	0.46
Unsaturated oils	354	693	3.28
Dairy fats	0	370	1.75
Lard or tallow	36	8	0.04
All sweeteners	120	555	2.63
**Total**	**2,503**	**21,121**	**100**

Appendix 2: Imputation and substitution for missing ICP prices

We identified two limitations of the ICP price data that could be remedied with imputation or substitution. First, in 38 mostly affluent countries there is no price reported for some starchy staples on the global list, such as white potatoes. These could be the least-cost source in that food group but were omitted from that country's ICP reports, so we imputed their value using prices from a neighboring country as detailed below. Second, 26 countries have no price reported for any food in the dark green leafy vegetable food group, and for those we substituted the price of another vegetable. Palm oil and lard or tallow are even more often missing, so for those we substituted the price of unsaturated oils. These steps are described in detail below.

Affluent countries with missing starchy staple prices

In affluent Western countries, plus Japan and Korea, prices for several kinds of starchy staples were not reported to the ICP. For example, price of wheat flour (typically the cheapest source of calories in Western countries) is missing for Norway, Sweden and the USA. Similarly, the price of Irish potato is missing for Czech Republic and the USA. To improve the accuracy of price data for starchy staples in the higher-income countries listed in Table S5, we imputed values by replacing the missing observations using the mean price for all starchy staples in the same geographical sub-region (see Table S5). These sub-regions contain nearby countries with similar food markets, and in most cases are in a free-trade agreement such as NAFTA or the EU which would equalize price and availability among countries within the region.

Table S5. Countries for which the price of one or more starchy staples was imputed

Sub-region	Country	Number of imputed prices
Alpine countries	Austria	3
Australasia	Australia	1
Australasia	New Zealand	4
Balkans	Albania	5
Balkans	Bosnia and Herzegovina	2
Balkans	Macedonia, FYR	7
Balkans	Montenegro	6
Baltic States	Estonia	1
Benelux countries	Belgium	1
Benelux countries	Luxembourg	1
Carpathian states	Belarus	4
Carpathian states	Czech Republic	7
Carpathian states	Hungary	7
Carpathian states	Poland	6
Carpathian states	Romania	8
Carpathian states	Serbia	6
Carpathian states	Slovakia	8
Carpathian states	Ukraine	4
Caucasus	Armenia	2
Caucasus	Azerbaijan	2
Central Asia	Kazakhstan	1
Central Asia	Kyrgyzstan	8
Central Asia	Moldova	3
Central Asia	Tajikistan	9
Korea & Japan	Japan	4
Korea & Japan	Korea, Rep.	1
Mediterranean countries	Italy	2
Mediterranean countries	Portugal	1
Mediterranean countries	Slovenia	1
Mediterranean countries	Turkey	3
Nordic countries	Denmark	1
Nordic countries	Finland	2
Nordic countries	Iceland	2
Nordic countries	Norway	4
Nordic countries	Sweden	4
North-America	Canada	2
North-America	Mexico	4
North-America	United States	7

Food groups with few price observations

As described in appendix 1, certain food groups such as the dark green vegetables, palm oil, and animal fats (lard or tallow) have relatively few price observations. In these food groups, we have countries for which not a single price observation was recorded; see table S6. For these countries, we substituted prices from the nearest food group as follows:

- Dark green vegetable with a food from the 'Other vegetables' food group.
- Palm oil, and lard or tallow, with an item from the 'Unsaturated oils' food group.

This substitution can also be described as merging the two food groups, so that the price retained is the lowest-cost source from the group for which at least item is locally available.

Table S6: Number of countries with no price observations in the food group

Food group	Serving size (kcal/day)	N	% of all countries
Rice, wheat, corn & other cereals	811	0	0
Potatoes and cassava	39	0	0
Dark green vegetables	23	26	16.4
Red & orange vegetables	30	0	0
Other vegetables	25	0	0
All fruits	126	0	0
Whole milk or derivatives	153	0	0
Beef, lamb & pork	30	0	0
Poultry, eggs & fish	121	0	0
Legumes, nuts & soy foods	575	0	0
Palm oil	60	98	61.6
Unsaturated oils	354	0	0
Dairy fats	0	n/a	n/a
Lard or tallow	36	151	95.0
All sweeteners	120	0	0

Appendix 3: Food composition tables

Food composition tables provide estimates of energy and nutrient content of food items. Countries typically develop their own food composition databases that are frequently updated. However, high-quality food composition data are missing in several countries (3). This is particularly the case in poor countries that lack sufficient resources to build and/or maintain them. This has prompted international organizations like the Food and Agriculture Organization of the United Nations (FAO) to develop international food composition tables. However, the FAO's International Network of Food Data Systems (INFOODS) database is still incomplete containing several missing estimates for key food items (3).

In the absence of high-quality international food composition tables, we used the United States Department of Agriculture (USDA) National Nutrient Database for Standard Reference 28 to obtain the edible portion and energy content of all food items in the ICP database. The latest available USDA database version contains more than 8,500 food items that are both in raw and processed form (4). The USDA food composition tables are constructed using standardized methods and are widely used for foods elsewhere in the world. Many regional and national organizations base their food composition tables to the USDA database (5).

The limited available evidence suggest a good agreement with the USDA and other carefully conducted databases, particularly for energy composition estimates (6, 7). Therefore, in the absence of high quality international food composition database, we rely on the USDA food composition tables to estimate the calorie content of the ICP food items. The EAT-*Lancet* commission (8) also relied on the USDA database to assess the nutritional adequacy of the EAT-*Lancet* reference diet.

The agreement with the food items in the ICP data and the food items in the USDA database is generally good. However, for certain fish, seafood and meat products the product descriptions in the ICP refer to whole animals (with bones) that do not have a corresponding item in the USDA product list. In these instances we obtained estimates of edible portions from FAO/INFOODS (9). For meat products (e.g. goat mixed cut/with bones or live lamb, mutton, turkey, and chicken this conversion was straightforward. For seafood, the ICP product descriptions were incomplete and the following assumptions were made:

1. We used the FAO uFish1.0, FAO West Africa, and Bangladesh food composition tables.
2. When the ICP did not specify how the fish was prepared (i.e. dried, smoked or fillet), we assumed the price referred to a raw whole fish.
3. We matched edible portions when there was a good match: i.e. catfish to catfish. If the ICP product was Africa or Asia-specific, we matched to West-Africa or Bangladesh FCTs.
4. When the product was a whole fish with no FCT info, we used the average edible portion of all measured fish varieties, which was 0.51 % (i.e. refusal fraction: 0.49).
5. For dried fish in Africa and Asia, we assumed that the edible portion was 1 (i.e. eaten with bones and skin).
6. For smoked fish, we used an edible portion of salmon, which was 0.75 (i.e. refusal fraction of 0.25).

Appendix 4: Additional details on Povcal data

The World Bank's PovcalNet system is based on 850 household surveys covering about 1.2 million randomly sampled households in 127 countries. From these surveys, PovcalNet computes an entire income distribution, not just the mean, allowing users to input any income level and calculate the number of people in the country that fall below this threshold. We used this feature to calculate the number of people whose incomes were below our diet costs in each country. For more information on the methodology used by PovcalNet, please visit http://iresearch.worldbank.org/PovcalNet/methodology.aspx.

Table S7 lists the 18 countries for which the Povcal data are missing (out of the 159 for which we estimate the cost of the EAT-*Lancet* reference diet), along with the 2017—population estimates. Most of the countries (14 out of 18) are high-income countries.

Table S7: Countries for which the Povcal data is missing

Country	Region	Income group	Population in 2017
Bahrain	Middle East & North Africa	High income	1,492,584
Brunei Darussalam	East Asia & Pacific	High income	428,697
Cambodia	East Asia & Pacific	Lower middle income	16,005,373
Cuba	Latin America & Caribbean	Upper middle income	11,484,636
Equatorial Guinea	Sub-Saharan Africa	Upper middle income	1,267,689
Hong Kong SAR, China	East Asia & Pacific	High income	7,391,700
Kuwait	Middle East & North Africa	High income	4,136,528
Macao SAR, China	East Asia & Pacific	High income	622,567
New Zealand	East Asia & Pacific	High income	4,793,900
Oman	Middle East & North Africa	High income	4,636,262
Qatar	Middle East & North Africa	High income	2,639,211
Saudi Arabia	Middle East & North Africa	High income	32,938,213
Singapore	East Asia & Pacific	High income	5,612,253
St. Kitts and Nevis	Latin America & Caribbean	High income	55,345
Suriname	Latin America & Caribbean	Upper middle income	563,402
Taiwan, China	East Asia & Pacific	High income	.
Trinidad and Tobago	Latin America & Caribbean	High income	1,369,125
United Arab Emirates	Middle East & North Africa	High income	9,400,145
		**Total**	**104,837,630**

The Povcal welfare estimate is based on consumption in 81 countries (57 % of the 141 countries in our data) and on income in 60 countries (43 %). Table S8 provides the data type for each country.

Table S8: Povcal data type (consumption or income) for each country

Country	Income group	Povcal data type
Australia	High income	income
Austria	High income	income
Belgium	High income	income
Canada	High income	income
Chile	High income	income
Croatia	High income	income
Cyprus	High income	income
Czech Republic	High income	income
Denmark	High income	income
Estonia	High income	income
Finland	High income	income
France	High income	income
Germany	High income	income
Greece	High income	income
Hungary	High income	income
Iceland	High income	income
Ireland	High income	income
Israel	High income	income
Italy	High income	income
Japan	High income	income
Korea, Rep.	High income	income
Latvia	High income	income
Lithuania	High income	income
Luxembourg	High income	income
Malta	High income	income
Netherlands	High income	income
Norway	High income	income
Poland	High income	income
Portugal	High income	income
Seychelles	High income	income
Slovakia	High income	income
Slovenia	High income	income
Spain	High income	income
Sweden	High income	income
Switzerland	High income	income
United Kingdom	High income	income
United States	High income	income
Uruguay	High income	income
Albania	Upper middle income	consumption

Country	Income group	Povcal data type
Algeria	Upper middle income	consumption
Angola	Upper middle income	consumption
Azerbaijan	Upper middle income	consumption
Belarus	Upper middle income	consumption
Bosnia and Herzegovina	Upper middle income	consumption
Botswana	Upper middle income	consumption
Brazil	Upper middle income	income
Bulgaria	Upper middle income	income
China	Upper middle income	consumption
Colombia	Upper middle income	income
Costa Rica	Upper middle income	income
Dominican Republic	Upper middle income	income
Ecuador	Upper middle income	income
Fiji	Upper middle income	consumption
Gabon	Upper middle income	consumption
Iraq	Upper middle income	consumption
Jamaica	Upper middle income	consumption
Jordan	Upper middle income	consumption
Kazakhstan	Upper middle income	consumption
Macedonia, FYR	Upper middle income	income
Malaysia	Upper middle income	income
Maldives	Upper middle income	consumption
Mauritius	Upper middle income	consumption
Mexico	Upper middle income	income
Montenegro	Upper middle income	consumption
Namibia	Upper middle income	consumption
Panama	Upper middle income	income
Paraguay	Upper middle income	income
Peru	Upper middle income	income
Romania	Upper middle income	income
Russian Federation	Upper middle income	consumption
Serbia	Upper middle income	income
South Africa	Upper middle income	consumption
Thailand	Upper middle income	consumption
Turkey	Upper middle income	consumption
Venezuela, RB	Upper middle income	income
Armenia	Lower middle income	consumption
Bangladesh	Lower middle income	consumption
Bhutan	Lower middle income	consumption
Bolivia	Lower middle income	income
Cameroon	Lower middle income	consumption
Cape Verde	Lower middle income	consumption
Congo, Rep.	Lower middle income	consumption

Country	Income group	Povcal data type
Côte d'Ivoire	Lower middle income	consumption
Djibouti	Lower middle income	consumption
Egypt, Arab Rep.	Lower middle income	consumption
El Salvador	Lower middle income	income
Ghana	Lower middle income	consumption
Guatemala	Lower middle income	income
Honduras	Lower middle income	income
India	Lower middle income	consumption
Indonesia	Lower middle income	consumption
Kenya	Lower middle income	consumption
Kyrgyzstan	Lower middle income	consumption
Lao PDR	Lower middle income	consumption
Lesotho	Lower middle income	consumption
Mauritania	Lower middle income	consumption
Moldova	Lower middle income	consumption
Mongolia	Lower middle income	consumption
Morocco	Lower middle income	consumption
Myanmar	Lower middle income	consumption
Nicaragua	Lower middle income	income
Nigeria	Lower middle income	consumption
Pakistan	Lower middle income	consumption
Palestinian Territory	Lower middle income	consumption
Philippines	Lower middle income	income
São Tomé and Principe	Lower middle income	consumption
Sri Lanka	Lower middle income	consumption
Sudan	Lower middle income	consumption
Swaziland	Lower middle income	consumption
Tajikistan	Lower middle income	consumption
Tunisia	Lower middle income	consumption
Ukraine	Lower middle income	consumption
Vietnam	Lower middle income	consumption
Yemen	Lower middle income	consumption
Zambia	Lower middle income	consumption
Benin	Low income	consumption
Burkina Faso	Low income	consumption
Burundi	Low income	consumption
Central African Republic	Low income	consumption
Chad	Low income	consumption
Comoros	Low income	consumption
Congo, Dem. Rep.	Low income	consumption
Ethiopia	Low income	consumption
Gambia, The	Low income	consumption
Guinea	Low income	consumption

Country	Income group	Povcal data type
Guinea-Bissau	Low income	consumption
Haiti	Low income	income
Liberia	Low income	consumption
Madagascar	Low income	consumption
Malawi	Low income	consumption
Mali	Low income	consumption
Mozambique	Low income	consumption
Nepal	Low income	consumption
Niger	Low income	consumption
Rwanda	Low income	consumption
Senegal	Low income	consumption
Sierra Leone	Low income	consumption
Tanzania	Low income	consumption
Togo	Low income	consumption
Uganda	Low income	consumption
Zimbabwe	Low income	consumption

Appendix 5: Affordability estimates based on daily per capita gross national income (GNI)

We also estimated affordability of the EAT-*Lancet* reference diet by comparing the estimated cost to the daily per capita gross national income (GNI) in 2011. GNI is sourced from the country's national accounts and it counts the total value of goods and services produced in the country, plus net income from abroad (10). Of note is that GNI tends to overstate mean income or consumption in the country (see 11, 12). As a result, affordability estimates based on daily per capita GNI are likely to be downward biased.

With this caveat in mind, Figures S1 and S2 below replicate Figures 2 and 3 in the manuscript using daily per capital GNI instead of daily per capita household consumption or income. The GNI-based affordability estimate is available for 156 countries (missing for Cuba, Djibouti and Taiwan).

The affordability of EAT-*Lancet* reference diets, as a fraction of daily per capita GNI, was an average of 2.7 % (IQR: 2·09–3·99) of daily per capita GNI in high-income countries, 9·8 % (IQR: 6·11-11·84) in upper-middle income countries, 24·1 % (IQR: 18·21–31·79) in lower-middle income countries, and 72.6 % (IQR: 48.73–83.11) in low-income countries (figure S1). Among regions, diet cost as a fraction of daily per capita GNI was lowest in North America (2.1 %; IQR: 1.92–2.35) and highest in sub-Saharan Africa (38·9 %; IQR: 23·39–73·63). There was considerable geographic variation even within regions (figure S2), and the estimated cost of an EAT-*Lancet* reference diet exceeded the entire daily per capita GNI in the three poorest African countries (Burundi, Democratic Republic of Congo, and Liberia).

Figure S1. Cost of the EAT-*Lancet* reference diet relative to daily per capita GNI by country income levels and major regions

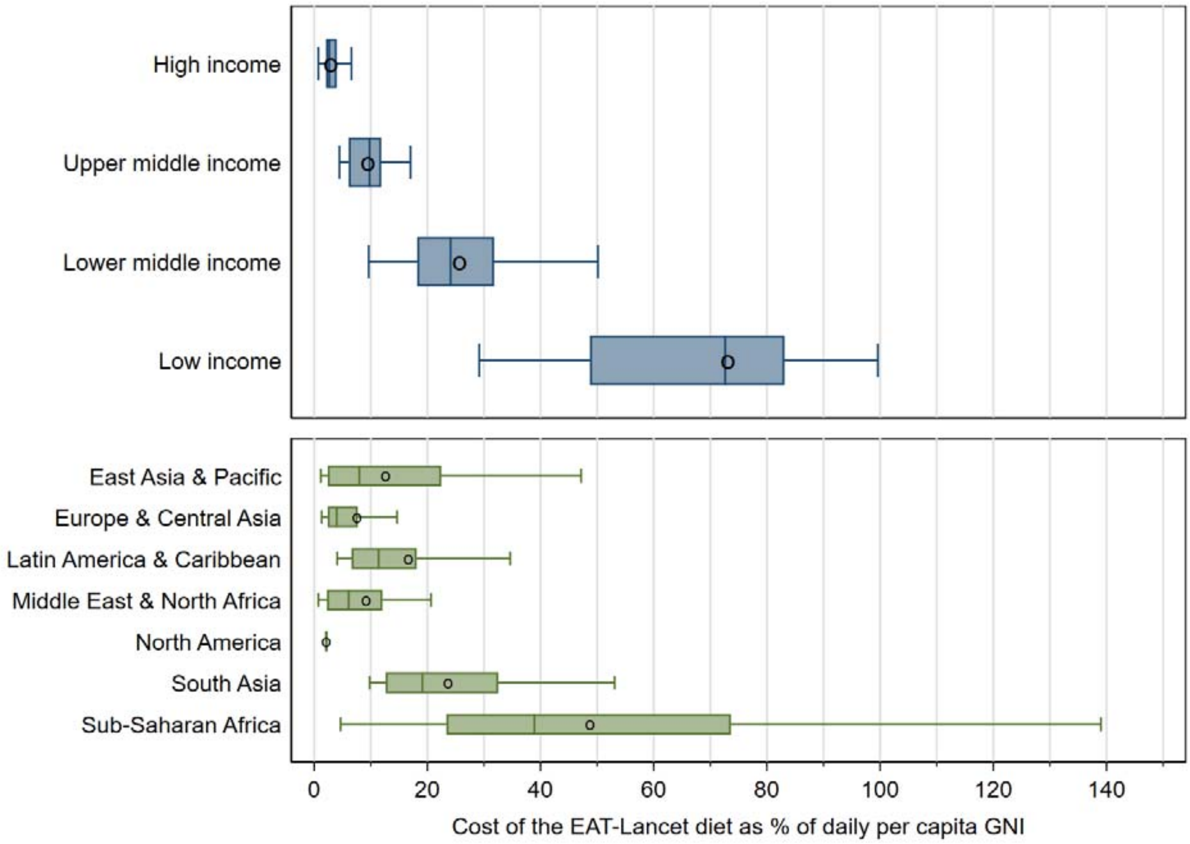

Note: We used price data from the International Comparison Program (ICP) to estimate the cost of the EAT-*Lancet* reference diet and compared these estimates to daily Gross National Income per capita. Data are percentages. The size of the box indicates the difference between the 25th percentile (the left-hand side of the box) and the 75th percentile (the right-hand side of the box) of the distribution. The bottom and top rule marks the bottom 5th and top 5th percentiles, respectively. The circle and the vertical bar rule inside the box show the mean and median value for the income group or geographic region, respectively. The vertical bar rule inside the box shows the median value for the income group or geographic region. N=156 countries; GNI estimates were not available for Cuba, Djibouti and Taiwan.

Figure S2. Cost of the EAT-*Lancet* reference diet relative to daily per capita gross national income (GNI)

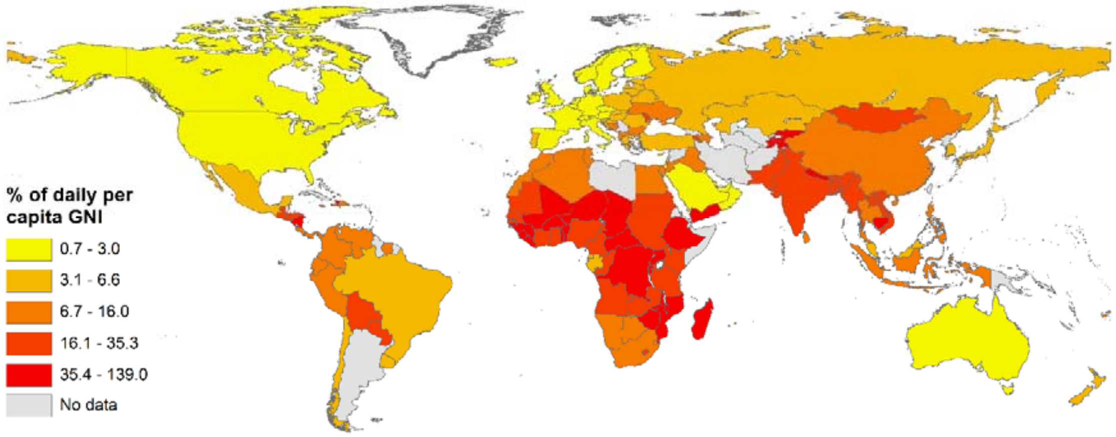

Note: We used price data from the International Comparison Program (ICP) to estimate the cost of the EAT-*Lancet* reference diet in 159 countries and compared these estimates to daily per capita gross national income. Estimates are reported in percentages. Countries are grouped by quintiles of the full cost relative to daily per capita GNI distribution. N=156 countries; GNI estimates were not available for Cuba, Djibouti and Taiwan.

Appendix 6: Cost of Nutrient Adequacy (CoNA)

Cost of Nutrient Adequacy (CoNA) is defined as the lowest cost of foods that meet all known requirements for essential nutrients and dietary energy requirements. Following the EAT-*Lancet* commission, we used a 60-kg woman who is 30 years of age as a reference. The linear programming approach considered the lower bound of the daily cost meeting both dietary energy needs of 2,503 calories, and DRIs for 20 nutrients, including the Acceptable Macronutrient Distribution Range (AMDR), the lower bounds of estimated average requirement (EAR) for nutrients as well as their upper limits (UL). The mathematical formula used to define CoNA is:

CoNA = min. $\{C = \Sigma_i p_i \times q_i\}$, subject to 6 constraints:

(1) $\Sigma_i a_{ij} \times q_i \geq EAR_j$

(2) $\Sigma_i a_{ij} \times qi \leq UL_j$

(3) $\Sigma_i a_{ij} \times q_i \leq AMDR_{j,upper} \times E / e_j$

(4) $\Sigma_i a_{ij} \times q_i \geq AMDR_{j,lower} \times E / e_j$

(5) $\Sigma_i a_{ie} \times q_i = E$

(6) $q_1 \geq 0, q_2 \geq 0, q_3 \geq 0, \ldots, qi \geq 0$

In this notation, the quantity of the jth nutrient in food i is denoted a_{ij}, which multiplied by its quantity consumed (q_i) must meet the population's estimated average requirement (EAR) meeting 50% of healthy people's needs, while remaining below upper limits (UL) for micronutrients and within a range for macronutrients determined by acceptable macronutrient distribution ranges ($AMDR_{lower}$ and $AMDR_{upper}$) as percentages of daily energy needs (E), at lowest total cost given all prices (p_i) within the further constraint of overall energy needs (E). The reference number e_j is the energy density of macronutrients, which is 4 kcal per gram of protein and carbohydrate and 9 kcal per gram of lipid. We included 20 nutrients for the CoNA calculation, including 3 macronutrients of protein, fat, carbohydrate, 7 minerals of calcium, iron, magnesium, phosphorous, zinc, copper and selenium, and 10 vitamins of vitamin C, thiamin, riboflavin, niacin, vitamin B6, folate, vitamin B12, vitamin A, retinol and vitamin E. We used the USDA National Nutrient Database (see appendix 3) to estimate the nutrient content of each ICP item. Finally, we used *lpSolve* package in R (13) for the linear programming calculations.

Appendix 7: World Bank's country classifications

The World Bank categorizes countries by geographic region and into four income groupings (high, upper-middle, lower-middle and low) according to their income level. The income levels are based on per capita gross national income (GNI). The income thresholds (as of July, 2018) used by the World Bank are (current US$):

- High-income > $12,055;
- Upper-middle income: $3,896–$12,055;
- Lower-middle income: $996–$3,895; and
- Low-income < $995.

Table S9 shows the World Bank country classifications for the 159 countries used in analysis.

Table S9: World Bank country classifications (N=159)

Country	Region	World Bank Income Classification
Albania	Europe & Central Asia	Upper middle income
Algeria	Middle East & North Africa	Upper middle income
Angola	Sub-Saharan Africa	Upper middle income
Armenia	Europe & Central Asia	Lower middle income
Australia	East Asia & Pacific	High income
Austria	Europe & Central Asia	High income
Azerbaijan	Europe & Central Asia	Upper middle income
Bahrain	Middle East & North Africa	High income
Bangladesh	South Asia	Lower middle income
Belarus	Europe & Central Asia	Upper middle income
Belgium	Europe & Central Asia	High income
Benin	Sub-Saharan Africa	Low income
Bhutan	South Asia	Lower middle income
Bolivia	Latin America & Caribbean	Lower middle income
Bosnia and Herzegovina	Europe & Central Asia	Upper middle income
Botswana	Sub-Saharan Africa	Upper middle income
Brazil	Latin America & Caribbean	Upper middle income
Brunei Darussalam	East Asia & Pacific	High income
Bulgaria	Europe & Central Asia	Upper middle income
Burkina Faso	Sub-Saharan Africa	Low income
Burundi	Sub-Saharan Africa	Low income
Cambodia	East Asia & Pacific	Lower middle income
Cameroon	Sub-Saharan Africa	Lower middle income
Canada	North America	High income
Cape Verde	Sub-Saharan Africa	Lower middle income
Central African Republic	Sub-Saharan Africa	Low income
Chad	Sub-Saharan Africa	Low income
Chile	Latin America & Caribbean	High income
China	East Asia & Pacific	Upper middle income

Country	Region	World Bank Income Classification
Colombia	Latin America & Caribbean	Upper middle income
Comoros	Sub-Saharan Africa	Low income
Congo, Dem. Rep.	Sub-Saharan Africa	Low income
Congo, Rep.	Sub-Saharan Africa	Lower middle income
Costa Rica	Latin America & Caribbean	Upper middle income
Côte d'Ivoire	Sub-Saharan Africa	Lower middle income
Croatia	Europe & Central Asia	High income
Cuba	Latin America & Caribbean	Upper middle income
Cyprus	Europe & Central Asia	High income
Czech Republic	Europe & Central Asia	High income
Denmark	Europe & Central Asia	High income
Djibouti	Middle East & North Africa	Lower middle income
Dominican Republic	Latin America & Caribbean	Upper middle income
Ecuador	Latin America & Caribbean	Upper middle income
Egypt, Arab Rep.	Middle East & North Africa	Lower middle income
El Salvador	Latin America & Caribbean	Lower middle income
Equatorial Guinea	Sub-Saharan Africa	Upper middle income
Estonia	Europe & Central Asia	High income
Ethiopia	Sub-Saharan Africa	Low income
Fiji	East Asia & Pacific	Upper middle income
Finland	Europe & Central Asia	High income
France	Europe & Central Asia	High income
Gabon	Sub-Saharan Africa	Upper middle income
Gambia, The	Sub-Saharan Africa	Low income
Germany	Europe & Central Asia	High income
Ghana	Sub-Saharan Africa	Lower middle income
Greece	Europe & Central Asia	High income
Guatemala	Latin America & Caribbean	Lower middle income
Guinea	Sub-Saharan Africa	Low income
Guinea-Bissau	Sub-Saharan Africa	Low income
Haiti	Latin America & Caribbean	Low income
Honduras	Latin America & Caribbean	Lower middle income
Hong Kong SAR, China	East Asia & Pacific	High income
Hungary	Europe & Central Asia	High income
Iceland	Europe & Central Asia	High income
India	South Asia	Lower middle income
Indonesia	East Asia & Pacific	Lower middle income
Iraq	Middle East & North Africa	Upper middle income
Ireland	Europe & Central Asia	High income
Israel	Middle East & North Africa	High income
Italy	Europe & Central Asia	High income
Jamaica	Latin America & Caribbean	Upper middle income
Japan	East Asia & Pacific	High income

Country	Region	World Bank Income Classification
Jordan	Middle East & North Africa	Upper middle income
Kazakhstan	Europe & Central Asia	Upper middle income
Kenya	Sub-Saharan Africa	Lower middle income
Korea, Rep.	East Asia & Pacific	High income
Kuwait	Middle East & North Africa	High income
Kyrgyzstan	Europe & Central Asia	Lower middle income
Lao PDR	East Asia & Pacific	Lower middle income
Latvia	Europe & Central Asia	High income
Lesotho	Sub-Saharan Africa	Lower middle income
Liberia	Sub-Saharan Africa	Low income
Lithuania	Europe & Central Asia	High income
Luxembourg	Europe & Central Asia	High income
Macao SAR, China	East Asia & Pacific	High income
Macedonia, FYR	Europe & Central Asia	Upper middle income
Madagascar	Sub-Saharan Africa	Low income
Malawi	Sub-Saharan Africa	Low income
Malaysia	East Asia & Pacific	Upper middle income
Maldives	South Asia	Upper middle income
Mali	Sub-Saharan Africa	Low income
Malta	Middle East & North Africa	High income
Mauritania	Sub-Saharan Africa	Lower middle income
Mauritius	Sub-Saharan Africa	Upper middle income
Mexico	Latin America & Caribbean	Upper middle income
Moldova	Europe & Central Asia	Lower middle income
Mongolia	East Asia & Pacific	Lower middle income
Montenegro	Europe & Central Asia	Upper middle income
Morocco	Middle East & North Africa	Lower middle income
Mozambique	Sub-Saharan Africa	Low income
Myanmar	East Asia & Pacific	Lower middle income
Namibia	Sub-Saharan Africa	Upper middle income
Nepal	South Asia	Low income
Netherlands	Europe & Central Asia	High income
New Zealand	East Asia & Pacific	High income
Nicaragua	Latin America & Caribbean	Lower middle income
Niger	Sub-Saharan Africa	Low income
Nigeria	Sub-Saharan Africa	Lower middle income
Norway	Europe & Central Asia	High income
Oman	Middle East & North Africa	High income
Palestinian Territory	Middle East & North Africa	Lower middle income
Pakistan	South Asia	Lower middle income
Panama	Latin America & Caribbean	Upper middle income
Paraguay	Latin America & Caribbean	Upper middle income
Peru	Latin America & Caribbean	Upper middle income

Country	Region	World Bank Income Classification
Philippines	East Asia & Pacific	Lower middle income
Poland	Europe & Central Asia	High income
Portugal	Europe & Central Asia	High income
Qatar	Middle East & North Africa	High income
Romania	Europe & Central Asia	Upper middle income
Russian Federation	Europe & Central Asia	Upper middle income
Rwanda	Sub-Saharan Africa	Low income
São Tomé and Principe	Sub-Saharan Africa	Lower middle income
Saudi Arabia	Middle East & North Africa	High income
Senegal	Sub-Saharan Africa	Low income
Serbia	Europe & Central Asia	Upper middle income
Seychelles	Sub-Saharan Africa	High income
Sierra Leone	Sub-Saharan Africa	Low income
Singapore	East Asia & Pacific	High income
Slovakia	Europe & Central Asia	High income
Slovenia	Europe & Central Asia	High income
South Africa	Sub-Saharan Africa	Upper middle income
Spain	Europe & Central Asia	High income
Sri Lanka	South Asia	Lower middle income
St. Kitts and Nevis	Latin America & Caribbean	High income
Sudan	Sub-Saharan Africa	Lower middle income
Suriname	Latin America & Caribbean	Upper middle income
Swaziland	Sub-Saharan Africa	Lower middle income
Sweden	Europe & Central Asia	High income
Switzerland	Europe & Central Asia	High income
Taiwan, China	East Asia & Pacific	High income
Tajikistan	Europe & Central Asia	Lower middle income
Tanzania	Sub-Saharan Africa	Low income
Thailand	East Asia & Pacific	Upper middle income
Togo	Sub-Saharan Africa	Low income
Trinidad and Tobago	Latin America & Caribbean	High income
Tunisia	Middle East & North Africa	Lower middle income
Turkey	Europe & Central Asia	Upper middle income
Uganda	Sub-Saharan Africa	Low income
Ukraine	Europe & Central Asia	Lower middle income
United Arab Emirates	Middle East & North Africa	High income
United Kingdom	Europe & Central Asia	High income
United States	North America	High income
Uruguay	Latin America & Caribbean	High income
Venezuela, RB	Latin America & Caribbean	Upper middle income
Vietnam	East Asia & Pacific	Lower middle income
Yemen	Middle East & North Africa	Lower middle income
Zambia	Sub-Saharan Africa	Lower middle income

Country	Region	World Bank Income Classification
Zimbabwe	Sub-Saharan Africa	Low income

Appendix 8: Cost of the EAT-Lancet reference diet relative to mean daily per capita household income, map

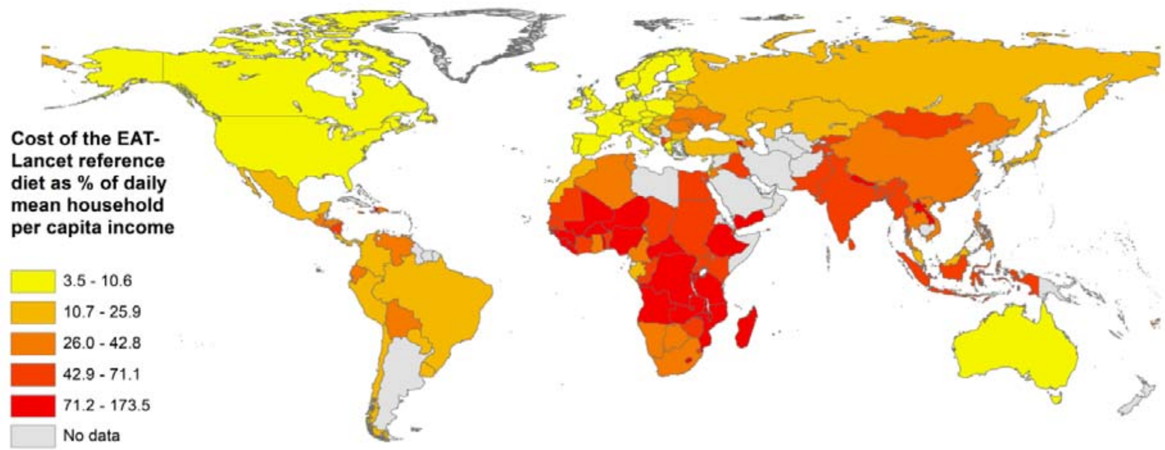

Figure S3. Cost of the EAT-*Lancet* reference diet relative to mean daily per capita household income

We used price data from the International Comparison Program (ICP) to estimate the cost of the EAT-*Lancet* diet in 141 countries and compared these estimates to mean daily per capita household consumption or income. Estimates are reported in percentages. Countries are grouped by quintiles of the full cost relative to mean daily per capita household consumption or income. N=141 countries.

Appendix 9: Composition of reference diets for EAT-*Lancet* and minimum cost of nutrient adequacy

Table S10. Composition of reference diets for EAT-*Lancet* and minimum cost of nutrient adequacy, by national income and geographic regions

Broad food group:	Starchy staples	Legumes & nuts	Fruits & vegetables	Dairy	Meat, eggs & fish	Oils & fats	Sweeteners	Total
**EAT-*Lancet* diet**	850	204	153	151	575	450	120	2,503
**Minimum cost of nutrient adequacy (CoNA)**								
Global	1,498	348	113	149	37	332	25	2,503
By country income level								
High income	1,631	227	35	206	10	387	6	2,503
Upper middle income	1,515	259	67	203	32	358	69	2,503
Lower middle income	1,422	497	144	92	26	299	23	2,503
Low income	1,324	498	290	42	113	236	0	2,503
By geographic region								
East Asia & Pacific	1,550	410	75	72	23	363	10	2,503
Europe & Central Asia	1,580	310	48	210	10	345	0	2,503
Latin America & Caribbean	1,312	210	72	303	48	406	152	2,503
Middle East & North Africa	1,730	214	66	121	11	361	0	2,503
North America	1,155	719	30	135	23	441	0	2,503
South Asia	1,771	205	74	146	6	262	39	2,503
Sub-Saharan Africa	1,380	482	241	56	79	265	0	2,503

Data show total dietary energy (kcal/day), by food group as specified for EAT-*Lancet* reference diets in the first row. Intake levels required to reach minimum cost of nutrient adequacy meet the same energy balance of 2,503 calories, and meet essential nutrient needs for a typical healthy woman specified as estimated average requirements, upper limits and average macronutrient distribution ranges for 20 essential nutrients. The nutrient-based diet may not meet requirements for other attributes specified in the EAT-*Lancet* food groups such as the phytochemicals in fruits & vegetables, or the bioavailability of nutrients in flesh foods. N=159 countries.

Table S3: ICP food items under each EAT food group

ICP product list	EAT-*Lancet* food group	ICP item name
Global	Rice, wheat, corn & other cereals	All-butter croissant
Global	Rice, wheat, corn & other cereals	Baguette
Global	Rice, wheat, corn & other cereals	Basmati Rice
Global	Rice, wheat, corn & other cereals	Brown rice - Family Pack
Global	Rice, wheat, corn & other cereals	Couscous
Global	Rice, wheat, corn & other cereals	Dried Noodles
Global	Rice, wheat, corn & other cereals	Instant Noodles
Global	Rice, wheat, corn & other cereals	Jasmine Rice
Global	Rice, wheat, corn & other cereals	Long grain rice - Family Pack
Global	Rice, wheat, corn & other cereals	Long grain rice - Non-Parboiled
Global	Rice, wheat, corn & other cereals	Long grain rice - Parboiled
Global	Rice, wheat, corn & other cereals	Macaroni
Global	Rice, wheat, corn & other cereals	Maize
Global	Rice, wheat, corn & other cereals	Maize Flour White
Global	Rice, wheat, corn & other cereals	Oats, rolled
Global	Rice, wheat, corn & other cereals	Pita bread
Global	Rice, wheat, corn & other cereals	Roll
Global	Rice, wheat, corn & other cereals	Salted crackers
Global	Rice, wheat, corn & other cereals	Short pasta
Global	Rice, wheat, corn & other cereals	Short-grained rice
Global	Rice, wheat, corn & other cereals	Sliced White bread
Global	Rice, wheat, corn & other cereals	Spaghetti
Global	Rice, wheat, corn & other cereals	Tinned sweet corn/Maize
Global	Rice, wheat, corn & other cereals	Vermicelli (Angel Hair)
Global	Rice, wheat, corn & other cereals	Wheat Semolina (Suji)
Global	Rice, wheat, corn & other cereals	Wheat flour, not self-rising
Global	Rice, wheat, corn & other cereals	White bread
Global	Rice, wheat, corn & other cereals	White rice, 25% broken
Global	Rice, wheat, corn & other cereals	White rice, Medium Grain
Global	Rice, wheat, corn & other cereals	Whole wheat bread
Asia	Rice, wheat, corn & other cereals	Bajra Flour
Asia	Rice, wheat, corn & other cereals	Beaten rice, Chira
Asia	Rice, wheat, corn & other cereals	Coarse #2
Asia	Rice, wheat, corn & other cereals	Coarse #3
Asia	Rice, wheat, corn & other cereals	Coarse #5
Asia	Rice, wheat, corn & other cereals	Coarse #6
Asia	Rice, wheat, corn & other cereals	Corn/Maize Flour, loose
Asia	Rice, wheat, corn & other cereals	Cream crackers
Asia	Rice, wheat, corn & other cereals	Egg noodles
Asia	Rice, wheat, corn & other cereals	Fresh rice noodles
Asia	Rice, wheat, corn & other cereals	Glutinous Rice
Asia	Rice, wheat, corn & other cereals	Instant noodles

ICP product list	EAT-*Lancet* food group	ICP item name
Asia	Rice, wheat, corn & other cereals	Maize
Asia	Rice, wheat, corn & other cereals	Millet, Sorghum
Asia	Rice, wheat, corn & other cereals	Premium rice #1
Asia	Rice, wheat, corn & other cereals	Premium rice #2
Asia	Rice, wheat, corn & other cereals	Premium rice #3
Asia	Rice, wheat, corn & other cereals	Rice flour
Asia	Rice, wheat, corn & other cereals	Roll or bun, loose
Asia	Rice, wheat, corn & other cereals	Roll or bun, prepacked
Asia	Rice, wheat, corn & other cereals	Sattu
Asia	Rice, wheat, corn & other cereals	Semolina, Suji
Asia	Rice, wheat, corn & other cereals	Snack crackers
Asia	Rice, wheat, corn & other cereals	Wheat flour, loose
Asia	Rice, wheat, corn & other cereals	White bread, unsliced loaf
Asia	Rice, wheat, corn & other cereals	White rice #1
Asia	Rice, wheat, corn & other cereals	White rice #10
Asia	Rice, wheat, corn & other cereals	White rice #3
Asia	Rice, wheat, corn & other cereals	White rice #4
Asia	Rice, wheat, corn & other cereals	White rice #5
Asia	Rice, wheat, corn & other cereals	White rice #6
Asia	Rice, wheat, corn & other cereals	White rice #7
Asia	Rice, wheat, corn & other cereals	White rice #9
Asia	Rice, wheat, corn & other cereals	White wheat
Asia	Rice, wheat, corn & other cereals	Wholemeal flour, Atta
Africa	Rice, wheat, corn & other cereals	Couscous (millet)
Africa	Rice, wheat, corn & other cereals	Flatbread
Africa	Rice, wheat, corn & other cereals	Lasagna (sheets)
Africa	Rice, wheat, corn & other cereals	Long-grained rice
Africa	Rice, wheat, corn & other cereals	Maize Flour Yellow
Africa	Rice, wheat, corn & other cereals	Maizena
Africa	Rice, wheat, corn & other cereals	Millet Flour
Africa	Rice, wheat, corn & other cereals	Millet Whole Grain
Africa	Rice, wheat, corn & other cereals	Round bread
Africa	Rice, wheat, corn & other cereals	Sliced brown bread
Africa	Rice, wheat, corn & other cereals	Sorghum Red Grains
Africa	Rice, wheat, corn & other cereals	Sorghum White Grains
Africa	Rice, wheat, corn & other cereals	Sweet Bread
Africa	Rice, wheat, corn & other cereals	White Maize grains
Africa	Rice, wheat, corn & other cereals	Yellow Broken Maize Grains
Africa	Rice, wheat, corn & other cereals	Yellow Maize Grains
Africa	Rice, wheat, corn & other cereals	Yellow Maize Grains, Branless
LAC	Rice, wheat, corn & other cereals	Brown rice - small pack
LAC	Rice, wheat, corn & other cereals	Corn tortilla
LAC	Rice, wheat, corn & other cereals	Long grain rice - loose

ICP product list	EAT-*Lancet* food group	ICP item name
LAC	Rice, wheat, corn & other cereals	Maize Flour White
LAC	Rice, wheat, corn & other cereals	Maize semolina
LAC	Rice, wheat, corn & other cereals	Pita bread
LAC	Rice, wheat, corn & other cereals	Roll
LAC	Rice, wheat, corn & other cereals	Short past with eggs
LAC	Rice, wheat, corn & other cereals	Short-grained rice - prepacked
LAC	Rice, wheat, corn & other cereals	Spaghetti, with eggs
LAC	Rice, wheat, corn & other cereals	Vermicelli (angel hair), with eggs
LAC	Rice, wheat, corn & other cereals	White rice, 20% broken
LAC	Rice, wheat, corn & other cereals	White rice, medium grain - prepacked
LAC	Rice, wheat, corn & other cereals	Yellow maize flour
West-Asia	Rice, wheat, corn & other cereals	Corn
West-Asia	Rice, wheat, corn & other cereals	Flat (Iranian) Bread
West-Asia	Rice, wheat, corn & other cereals	Hard Loose Bulgur
West-Asia	Rice, wheat, corn & other cereals	Kiln Bread
West-Asia	Rice, wheat, corn & other cereals	Lasagna
West-Asia	Rice, wheat, corn & other cereals	Rice [Specified brand]
West-Asia	Rice, wheat, corn & other cereals	Soft/ Loose Bulgur
West-Asia	Rice, wheat, corn & other cereals	Thailand Rice
West-Asia	Rice, wheat, corn & other cereals	Toast petit beurre brown crunchy
Global	Potatoes and cassava	Brown Potatoes
Global	Potatoes and cassava	Cassava - Manioc - Yuka
Global	Potatoes and cassava	Sweet Potatoes
Asia	Potatoes and cassava	Taro
Asia	Potatoes and cassava	White potato
Africa	Potatoes and cassava	Green Plantain
Africa	Potatoes and cassava	Whole Cassava
LAC	Potatoes and cassava	Malanga / yautia / tannia / tannier / macabo
Global	Dark green vegetables	Spinach
Asia	Dark green vegetables	Spinach Chinese
Asia	Dark green vegetables	Water Spinach
Africa	Dark green vegetables	Cassava Leaves
Africa	Dark green vegetables	Chives
Africa	Dark green vegetables	Pumpkin leaves
Africa	Dark green vegetables	Rape Leaves
Africa	Dark green vegetables	Sorrel Leaves
Africa	Dark green vegetables	Spinach
Africa	Dark green vegetables	Sweet Potato Leaves
Africa	Dark green vegetables	Taro Leaves
Global	Red & orange vegetables	Bell pepper
Global	Red & orange vegetables	Carrots
Global	Red & orange vegetables	Round tomato, loose
Asia	Red & orange vegetables	Pumpkin

ICP product list	EAT-*Lancet* food group	ICP item name
Africa	Red & orange vegetables	Beetroots
Africa	Red & orange vegetables	Peeled Tomatoes
LAC	Red & orange vegetables	Beetroot
LAC	Red & orange vegetables	Buttercup squash
LAC	Red & orange vegetables	Plum tomatoes
West-Asia	Red & orange vegetables	Domestic Beetroot
West-Asia	Red & orange vegetables	Domestic Carrots
West-Asia	Red & orange vegetables	Domestic Tomatoes
West-Asia	Red & orange vegetables	Imported Beetroot
West-Asia	Red & orange vegetables	Imported Tomatoes
Global	Other vegetables	Cauliflower
Global	Other vegetables	Cucumber
Global	Other vegetables	Eggplant (aubergine)
Global	Other vegetables	Green cabbage
Global	Other vegetables	Lettuce
Global	Other vegetables	Onion
Global	Other vegetables	Tinned Button Mushrooms
Global	Other vegetables	Tinned green peas
Asia	Other vegetables	Mushrooms, dried
Asia	Other vegetables	Radish, white
Africa	Other vegetables	Broccoli
Africa	Other vegetables	Celery
Africa	Other vegetables	Dried Okra
Africa	Other vegetables	Fresh Okra
Africa	Other vegetables	Gherkins
Africa	Other vegetables	Green Asparagus
Africa	Other vegetables	Green Beans
Africa	Other vegetables	Mushrooms
Africa	Other vegetables	Radish
Africa	Other vegetables	Round Onions, red
Africa	Other vegetables	Spring Onions
Africa	Other vegetables	Turnips
LAC	Other vegetables	Celery
LAC	Other vegetables	Zucchini
West-Asia	Other vegetables	Cucumber Pickles
West-Asia	Other vegetables	Domestic Green beans (Pulses)
West-Asia	Other vegetables	Domestic Green bell peppers
West-Asia	Other vegetables	Domestic Okra
West-Asia	Other vegetables	Domestic Red bell peppers
West-Asia	Other vegetables	Domestic Round red radish
West-Asia	Other vegetables	Domestic White onion
West-Asia	Other vegetables	Domestic Zucchini
West-Asia	Other vegetables	Green beans

ICP product list	EAT-*Lancet* food group	ICP item name
West-Asia	Other vegetables	Imported Green beans (Pulses)
West-Asia	Other vegetables	Imported Okra
West-Asia	Other vegetables	Imported Red bell peppers
West-Asia	Other vegetables	Imported White onion
West-Asia	Other vegetables	Imported Zucchini
West-Asia	Other vegetables	Okra
West-Asia	Other vegetables	Vegetarian vegetable soup
Global	All fruits	Apple, Red Delicious
Global	All fruits	Apple, Typical Local Variety
Global	All fruits	Avocado
Global	All fruits	Banana, Standard
Global	All fruits	Dried dates
Global	All fruits	Grapefruit
Global	All fruits	Grapes, green
Global	All fruits	Lemon
Global	All fruits	Mango
Global	All fruits	Melon
Global	All fruits	Orange
Global	All fruits	Papaya
Global	All fruits	Peach
Global	All fruits	Pineapple
Global	All fruits	Tinned pineapple
Global	All fruits	Watermelon
Asia	All fruits	Grapes, violet, with seed
Asia	All fruits	Lime
Africa	All fruits	Banana, short finger length
Africa	All fruits	Clementine
Africa	All fruits	Dried apricots
Africa	All fruits	Dried plums
Africa	All fruits	Grapes, red
Africa	All fruits	Large Mango (Grafted)
Africa	All fruits	Passion fruit
Africa	All fruits	Tinned peaches
LAC	All fruits	Canned peach halves
LAC	All fruits	Dark raisins
LAC	All fruits	Melon, Honeydew
LAC	All fruits	Passion fruit
West-Asia	All fruits	Apple
West-Asia	All fruits	Apricots
West-Asia	All fruits	Domestic Apricots
West-Asia	All fruits	Domestic Cherries
West-Asia	All fruits	Domestic Figs
West-Asia	All fruits	Domestic Grapes

ICP product list	EAT-*Lancet* food group	ICP item name
West-Asia	All fruits	Domestic Guava
West-Asia	All fruits	Domestic Mango
West-Asia	All fruits	Domestic Pears
West-Asia	All fruits	Domestic Plums
West-Asia	All fruits	Domestic Pomegranate
West-Asia	All fruits	Domestic Red Dates
West-Asia	All fruits	Domestic Strawberries
West-Asia	All fruits	Domestic Dates
West-Asia	All fruits	Figs
West-Asia	All fruits	Fruit cocktail, Canned
West-Asia	All fruits	Imported Apricots
West-Asia	All fruits	Imported Cherries
West-Asia	All fruits	Imported Coconut
West-Asia	All fruits	Imported Dates
West-Asia	All fruits	Imported Figs
West-Asia	All fruits	Imported Grapes
West-Asia	All fruits	Imported Guava
West-Asia	All fruits	Imported Kiwi
West-Asia	All fruits	Imported Melon
West-Asia	All fruits	Imported Pears, Premium
West-Asia	All fruits	Imported Plums
West-Asia	All fruits	Imported Pomegranate
West-Asia	All fruits	Imported Red Dates
West-Asia	All fruits	Orange
West-Asia	All fruits	Orange
Global	Whole milk or derivatives	Cheese, Camembert Type
Global	Whole milk or derivatives	Cheese, Cheddar
Global	Whole milk or derivatives	Cheese, Gouda Type
Global	Whole milk or derivatives	Cheese, processed
Global	Whole milk or derivatives	Cream cheese
Global	Whole milk or derivatives	Milk, low-fat, Pasteurized
Global	Whole milk or derivatives	Milk, powdered
Global	Whole milk or derivatives	Milk, un-skimmed Pasteurized
Global	Whole milk or derivatives	Milk, un-skimmed UHT
Global	Whole milk or derivatives	Sour cream
Global	Whole milk or derivatives	Yoghurt, plain
Asia	Whole milk or derivatives	Buffalo milk, not pasteurized
Asia	Whole milk or derivatives	Buffalo milk, pasteurized
Asia	Whole milk or derivatives	Cheese spread
Asia	Whole milk or derivatives	Local cheese
Asia	Whole milk or derivatives	Local curd
Asia	Whole milk or derivatives	Milk, not pasteurized
Asia	Whole milk or derivatives	Powdered milk, box

ICP product list	EAT-*Lancet* food group	ICP item name
Asia	Whole milk or derivatives	Yoghurt drink
Asia	Whole milk or derivatives	Yoghurt, fruit
Africa	Whole milk or derivatives	Crème fraîche
Africa	Whole milk or derivatives	Fresh cheese edam
Africa	Whole milk or derivatives	Fresh cheese Emmental
Africa	Whole milk or derivatives	Liquid Yoghurt
Africa	Whole milk or derivatives	Powdered milk
Africa	Whole milk or derivatives	Sour (clotted) milk
Africa	Whole milk or derivatives	Yoghurt with natural fruits
LAC	Whole milk or derivatives	Cheese, mozzarella type
LAC	Whole milk or derivatives	Fresh cheese
LAC	Whole milk or derivatives	Milk, low - fat , pasteurized in plastic bag
LAC	Whole milk or derivatives	Milk, low-fat, UHT
LAC	Whole milk or derivatives	Powdered milk, in bag or box
LAC	Whole milk or derivatives	Yoghurt, with flavor
LAC	Whole milk or derivatives	Yogurt drink
West-Asia	Whole milk or derivatives	Cheese, Cottage (halloumi)
West-Asia	Whole milk or derivatives	Cheese, Feta
West-Asia	Whole milk or derivatives	Cheese, Haloumi
West-Asia	Whole milk or derivatives	Cheese, Kashkaval
West-Asia	Whole milk or derivatives	Cheese, Mozzarella
West-Asia	Whole milk or derivatives	Fresh Milk, 0% (import)
West-Asia	Whole milk or derivatives	Fresh Milk, 1.5-2.5%
West-Asia	Whole milk or derivatives	Fresh Milk, 3-4%
West-Asia	Whole milk or derivatives	Fresh Milk, 3-4%
West-Asia	Whole milk or derivatives	Fresh wet cheese
West-Asia	Whole milk or derivatives	Labneh
West-Asia	Whole milk or derivatives	Light whipping or whipping
West-Asia	Whole milk or derivatives	Local hard, dry cheese
Global	Beef, lamb & pork	100% Beef, minced
Global	Beef, lamb & pork	Bacon, smoked
Global	Beef, lamb & pork	Beef liver
Global	Beef, lamb & pork	Beef with bones
Global	Beef, lamb & pork	Beef, Center brisket
Global	Beef, lamb & pork	Beef, Fillet
Global	Beef, lamb & pork	Beef, Rump steak
Global	Beef, lamb & pork	Beef, for stew or curry
Global	Beef, lamb & pork	Corned beef
Global	Beef, lamb & pork	Goat mixed cut/with bones (non-refrigerated)
Global	Beef, lamb & pork	Lamb chops
Global	Beef, lamb & pork	Lamb whole leg
Global	Beef, lamb & pork	Mutton mixed cut
Global	Beef, lamb & pork	Pork ham, pressed

ICP product list	EAT-*Lancet* food group	ICP item name
Global	Beef, lamb & pork	Pork, fillet
Global	Beef, lamb & pork	Pork, loin chop
Global	Beef, lamb & pork	Pork, ribs
Global	Beef, lamb & pork	Pork, shoulder
Global	Beef, lamb & pork	Veal breast (non-refrigerated), with bones
Global	Beef, lamb & pork	Veal chops
Asia	Beef, lamb & pork	Bacon, pork
Asia	Beef, lamb & pork	Beef, Fillet, frozen
Asia	Beef, lamb & pork	Beef, with bones, non-specific cut
Asia	Beef, lamb & pork	Beef, without bones, non-specific cut
Asia	Beef, lamb & pork	Canned beef, chunks
Asia	Beef, lamb & pork	Goat leg
Asia	Beef, lamb & pork	Mutton chops
Asia	Beef, lamb & pork	Mutton/goat liver
Asia	Beef, lamb & pork	Pork liver
Asia	Beef, lamb & pork	Pork loin, without bones
Asia	Beef, lamb & pork	Pork thigh, with bones
Asia	Beef, lamb & pork	Pork, with bones, non-specific cut
Asia	Beef, lamb & pork	Pork, without bones, non-specific cut
Asia	Beef, lamb & pork	Round steak
Asia	Beef, lamb & pork	Sirloin steak
Asia	Beef, lamb & pork	Sliced ham, pork
Asia	Beef, lamb & pork	Veal, with bones
Africa	Beef, lamb & pork	Beef Feet/Trotters (Uncleaned)
Africa	Beef, lamb & pork	Beef Merguez (spiced)
Africa	Beef, lamb & pork	Beef ham
Africa	Beef, lamb & pork	Beef prepacked
Africa	Beef, lamb & pork	Beef without bones
Africa	Beef, lamb & pork	Lamb
Africa	Beef, lamb & pork	Live Goat
Africa	Beef, lamb & pork	Live Sheep
Africa	Beef, lamb & pork	Mutton Tripes
Africa	Beef, lamb & pork	Mutton chop
Africa	Beef, lamb & pork	Oxtail
Africa	Beef, lamb & pork	Pork meat
Africa	Beef, lamb & pork	Sirloin Steak
Africa	Beef, lamb & pork	Veal without offal
LAC	Beef, lamb & pork	Flank or skirt beef, for shredding
LAC	Beef, lamb & pork	Mortadella, loose
LAC	Beef, lamb & pork	Mortadella, prepacked
LAC	Beef, lamb & pork	Pork and beef sausages
LAC	Beef, lamb & pork	Pork ham, pressed, bulk or loose
West-Asia	Beef, lamb & pork	Domestic Fillet Steak(excluding round &sirloin)

ICP product list	EAT-*Lancet* food group	ICP item name
West-Asia	Beef, lamb & pork	Domestic Ground mutton (Fresh)
West-Asia	Beef, lamb & pork	Fillet (Round or sirloin)
West-Asia	Beef, lamb & pork	Goat, boneless. Non-specific cut
West-Asia	Beef, lamb & pork	Ground beef (Frozen)
West-Asia	Beef, lamb & pork	Imported Fillet Steak(excluding round &sirloin)
West-Asia	Beef, lamb & pork	Imported Ground Mutton (Fresh)
West-Asia	Beef, lamb & pork	Lamb (Fresh) with bones , Non-specific cut
West-Asia	Beef, lamb & pork	Lamb (Fresh), boneless, Non-specific cut
West-Asia	Beef, lamb & pork	Live lamb
West-Asia	Beef, lamb & pork	Live mutton
West-Asia	Beef, lamb & pork	Mutton Liver (Chilled)
West-Asia	Beef, lamb & pork	Mutton Liver (Fresh)
West-Asia	Beef, lamb & pork	Mutton with bones (Refrigerated) , Non-specific cut
West-Asia	Beef, lamb & pork	Veal with bones (Refrigerated)
West-Asia	Beef, lamb & pork	Veal, Boneless (Fresh unchilled)
West-Asia	Beef, lamb & pork	Veal, Boneless (Refrigerated)
Global	Poultry, eggs & fish	Black Pomfret
Global	Poultry, eggs & fish	Canned chicken
Global	Poultry, eggs & fish	Canned mackerel fillet in vegetable oil
Global	Poultry, eggs & fish	Canned sardine with skin
Global	Poultry, eggs & fish	Canned tuna without skin
Global	Poultry, eggs & fish	Carp
Global	Poultry, eggs & fish	Chicken breast with skin and bones
Global	Poultry, eggs & fish	Chicken breast without skin
Global	Poultry, eggs & fish	Chicken legs
Global	Poultry, eggs & fish	Cod (Gadus morhua)
Global	Poultry, eggs & fish	Dried Shrimp
Global	Poultry, eggs & fish	Large size chicken eggs
Global	Poultry, eggs & fish	Live chicken
Global	Poultry, eggs & fish	Mackerel, un-cleaned
Global	Poultry, eggs & fish	Medium size chicken eggs
Global	Poultry, eggs & fish	Mullet
Global	Poultry, eggs & fish	Red snapper
Global	Poultry, eggs & fish	Sea Bass
Global	Poultry, eggs & fish	Sea Crab
Global	Poultry, eggs & fish	Shrimps
Global	Poultry, eggs & fish	Smoked salmon
Global	Poultry, eggs & fish	Squid
Global	Poultry, eggs & fish	Tilapia
Global	Poultry, eggs & fish	Tuna steaks
Global	Poultry, eggs & fish	Whole Shrimps
Global	Poultry, eggs & fish	Whole chicken
Global	Poultry, eggs & fish	Whole chicken - Broiler

ICP product list	EAT-*Lancet* food group	ICP item name
Asia	Poultry, eggs & fish	Breakfast sausage, chicken
Asia	Poultry, eggs & fish	Catfish
Asia	Poultry, eggs & fish	Chicken drumsticks
Asia	Poultry, eggs & fish	Chicken egg, 1
Asia	Poultry, eggs & fish	Chicken eggs, 10, loose
Asia	Poultry, eggs & fish	Chicken wings
Asia	Poultry, eggs & fish	Chicken, non-specific cuts, frozen
Asia	Poultry, eggs & fish	Chicken, non-specific cuts, not frozen
Asia	Poultry, eggs & fish	Duck, whole
Asia	Poultry, eggs & fish	Fish ball
Asia	Poultry, eggs & fish	Fresh whole chicken
Asia	Poultry, eggs & fish	Mud Crab
Asia	Poultry, eggs & fish	Native house chicken
Asia	Poultry, eggs & fish	Prawn/Shrimp, medium
Asia	Poultry, eggs & fish	Prawn/Shrimp, small
Asia	Poultry, eggs & fish	Salted & semi-dried fish
Asia	Poultry, eggs & fish	Salted duck egg
Asia	Poultry, eggs & fish	Sea Lobster
Asia	Poultry, eggs & fish	Small fresh fish
Asia	Poultry, eggs & fish	Smoked fish
Asia	Poultry, eggs & fish	Sole
Asia	Poultry, eggs & fish	Spanish Mackerel
Asia	Poultry, eggs & fish	Squid, small
Asia	Poultry, eggs & fish	Tuna
Asia	Poultry, eggs & fish	Tuna Steak
Asia	Poultry, eggs & fish	White Pomfret
Africa	Poultry, eggs & fish	Anchovy
Africa	Poultry, eggs & fish	Bream
Africa	Poultry, eggs & fish	Capitaine
Africa	Poultry, eggs & fish	Catfish
Africa	Poultry, eggs & fish	Chicken wings
Africa	Poultry, eggs & fish	Dried Machoiron
Africa	Poultry, eggs & fish	Dried bonga
Africa	Poultry, eggs & fish	Dried sardines
Africa	Poultry, eggs & fish	Dried small fish
Africa	Poultry, eggs & fish	Duck - Dressed
Africa	Poultry, eggs & fish	Eggs, traditional production
Africa	Poultry, eggs & fish	Fresh Small Sardines
Africa	Poultry, eggs & fish	Frozen Capitaine in Sea Water
Africa	Poultry, eggs & fish	Frozen Nile Perch
Africa	Poultry, eggs & fish	Frozen Sea-bream
Africa	Poultry, eggs & fish	Frozen Shrimps
Africa	Poultry, eggs & fish	Frozen Whiting

ICP product list	EAT-*Lancet* food group	ICP item name
Africa	Poultry, eggs & fish	Giant shrimps
Africa	Poultry, eggs & fish	Grouper
Africa	Poultry, eggs & fish	Live Turkey
Africa	Poultry, eggs & fish	Lobster, chilled
Africa	Poultry, eggs & fish	Mackerel in vegetable oil
Africa	Poultry, eggs & fish	Mix Frozen Chicken Parts
Africa	Poultry, eggs & fish	Nile Perch
Africa	Poultry, eggs & fish	Red Snapper
Africa	Poultry, eggs & fish	Red mullet
Africa	Poultry, eggs & fish	Sardines in tomato sauce
Africa	Poultry, eggs & fish	Smoked carp
Africa	Poultry, eggs & fish	Smoked kapenta
Africa	Poultry, eggs & fish	Smoked kingfish
Africa	Poultry, eggs & fish	Smoked mboto
Africa	Poultry, eggs & fish	Smoked shrimps/prawns
Africa	Poultry, eggs & fish	Sole fish
Africa	Poultry, eggs & fish	Traditionally bred live chicken
Africa	Poultry, eggs & fish	Tuna
Africa	Poultry, eggs & fish	Tuna in vegetable oil
Africa	Poultry, eggs & fish	Turkey breast
LAC	Poultry, eggs & fish	Canned sardines with skin, in tomato sauce
LAC	Poultry, eggs & fish	Hake fillet
LAC	Poultry, eggs & fish	Maigre
LAC	Poultry, eggs & fish	Maigre fillet
LAC	Poultry, eggs & fish	Poultry sausages (chicken or turkey)
LAC	Poultry, eggs & fish	Red porgy
LAC	Poultry, eggs & fish	Red porgy fillet
LAC	Poultry, eggs & fish	Salted dry cod
LAC	Poultry, eggs & fish	Sardines
LAC	Poultry, eggs & fish	Surubi fillet
LAC	Poultry, eggs & fish	Tilapia fillet
West-Asia	Poultry, eggs & fish	Canned Tuna/Water
West-Asia	Poultry, eggs & fish	Caviar
West-Asia	Poultry, eggs & fish	Chicken soup
West-Asia	Poultry, eggs & fish	Chicken wings
West-Asia	Poultry, eggs & fish	Emperor fish
West-Asia	Poultry, eggs & fish	Giant Shrimp
West-Asia	Poultry, eggs & fish	Grouper (Hamour) fish
West-Asia	Poultry, eggs & fish	Processed shrimp
West-Asia	Poultry, eggs & fish	Tuna fish fresh
West-Asia	Poultry, eggs & fish	White or brown chicken eggs, Small size [domestic]
West-Asia	Poultry, eggs & fish	Whole chicken (Frozen)
West-Asia	Poultry, eggs & fish	Zubaida Fish

ICP product list	EAT-*Lancet* food group	ICP item name
Global	Legumes, nuts & soy foods	Bean Curd - Tofu
Global	Legumes, nuts & soy foods	Dried almonds
Global	Legumes, nuts & soy foods	Dried white beans
Global	Legumes, nuts & soy foods	Green/Mung Beans, dried
Global	Legumes, nuts & soy foods	Lentils, Dry
Global	Legumes, nuts & soy foods	Roasted groundnuts/peanuts
Global	Legumes, nuts & soy foods	Tinned white beans in tomato sauce
Asia	Legumes, nuts & soy foods	Dhal, Khesari
Asia	Legumes, nuts & soy foods	Dhal, Musur
Asia	Legumes, nuts & soy foods	Dhal, Split Peas
Asia	Legumes, nuts & soy foods	Moong dahl, loose
Asia	Legumes, nuts & soy foods	Peanuts in shell
Africa	Legumes, nuts & soy foods	Broad Beans
Africa	Legumes, nuts & soy foods	Caramel groundnuts
Africa	Legumes, nuts & soy foods	Cashew nuts
Africa	Legumes, nuts & soy foods	Macadamia nuts
Africa	Legumes, nuts & soy foods	Natural Groundnuts
Africa	Legumes, nuts & soy foods	Packed Peas
Africa	Legumes, nuts & soy foods	Peas
Africa	Legumes, nuts & soy foods	Peas
Africa	Legumes, nuts & soy foods	Pigeon peas
Africa	Legumes, nuts & soy foods	Roasted groundnuts
Africa	Legumes, nuts & soy foods	Spotted beans
LAC	Legumes, nuts & soy foods	Cashew
LAC	Legumes, nuts & soy foods	Dried black beans
LAC	Legumes, nuts & soy foods	Dried red beans
West-Asia	Legumes, nuts & soy foods	Almonds, Unhusked
West-Asia	Legumes, nuts & soy foods	Domestic Broad beans (Pulses)
West-Asia	Legumes, nuts & soy foods	Domestic Peanuts
West-Asia	Legumes, nuts & soy foods	Domestic Peas
West-Asia	Legumes, nuts & soy foods	Hazelnuts
West-Asia	Legumes, nuts & soy foods	Imported Broad beans (Pulses)
West-Asia	Legumes, nuts & soy foods	Imported Peanuts
West-Asia	Legumes, nuts & soy foods	Imported Peas
West-Asia	Legumes, nuts & soy foods	Imported Peas
West-Asia	Legumes, nuts & soy foods	Peas, Tinned
West-Asia	Legumes, nuts & soy foods	Walnuts
Global	Palm oil	Palm oil
Africa	Palm oil	Palm oil unrefined
West-Asia	Palm oil	Palm oil
Global	Unsaturated oils	Olive oil
Global	Unsaturated oils	Peanut oil
Global	Unsaturated oils	Soybean oil

ICP product list	EAT-*Lancet* food group	ICP item name
Global	Unsaturated oils	Sunflower oil
Global	Unsaturated oils	Vegetable oil
Asia	Unsaturated oils	Coconut oil
Asia	Unsaturated oils	Corn oil
Asia	Unsaturated oils	Mustard oil
Asia	Unsaturated oils	Olive oil, standard
Africa	Unsaturated oils	Maize oil
Africa	Unsaturated oils	Sesame oil
West-Asia	Unsaturated oils	Maize oil
West-Asia	Unsaturated oils	Olive Oil
West-Asia	Unsaturated oils	Pure Sesame Oil
West-Asia	Unsaturated oils	Sesame oil or oil Alserg
Global	Dairy fats	Butter, unsalted
Global	Dairy fats	Salted Butter
Asia	Dairy fats	Ghee
Asia	Dairy fats	Ghee, cow/buffalo
Africa	Dairy fats	Butter, sold loose
Africa	Dairy fats	Ghee
West-Asia	Dairy fats	Ghee
West-Asia	Lard or tallow	Animal Fats
Global	All sweeteners	Brown sugar
Global	All sweeteners	Natural honey, Mixed blossoms
Global	All sweeteners	White sugar
Asia	All sweeteners	White sugar, loose
Africa	All sweeteners	Brown sugar cubes
Africa	All sweeteners	Packed Brown sugar
Africa	All sweeteners	Packed White sugar
Africa	All sweeteners	Powdered Glucose
LAC	All sweeteners	White sugar, bulk
LAC	All sweeteners	White sugar, family size pack
West-Asia	All sweeteners	Processed honey, pure
West-Asia	All sweeteners	Processed honey, pure
West-Asia	All sweeteners	White sugar, granulated, cane
Global	Uncategorized	Apple juice
Global	Uncategorized	Baby cereals
Global	Uncategorized	Baby food
Global	Uncategorized	Black Pepper, ground
Global	Uncategorized	Butter biscuits
Global	Uncategorized	Carbonated Soft Drink [Specified brands] (Large)
Global	Uncategorized	Carbonated Soft Drink [Specified brands] (Small)
Global	Uncategorized	Chicken Extract (bouillon/stock cube)
Global	Uncategorized	Chili powder
Global	Uncategorized	Chili sauce

ICP product list	EAT-*Lancet* food group	ICP item name
Global	Uncategorized	Chilies (Long)
Global	Uncategorized	Chocolate bar
Global	Uncategorized	Chocolate cake (Individual serving)
Global	Uncategorized	Chocolate cake (Whole)
Global	Uncategorized	Cocoa Powder, Tin
Global	Uncategorized	Coffee Roasted 100% Arabica
Global	Uncategorized	Coffee Roasted 100% Robusta
Global	Uncategorized	Cooking salt
Global	Uncategorized	Cornflakes [Specified brand]
Global	Uncategorized	Curry Powder
Global	Uncategorized	Flavored biscuits/cookies sweet
Global	Uncategorized	Frozen chipped potatoes
Global	Uncategorized	Fruit drops (Hard candies)
Global	Uncategorized	Garlic (White)
Global	Uncategorized	Ginger (Mature)
Global	Uncategorized	Green Olives (with stones)
Global	Uncategorized	Ice cream, Cornetto-type
Global	Uncategorized	Ice cream, packed
Global	Uncategorized	Instant coffee [Specified brand]
Global	Uncategorized	Lemonade
Global	Uncategorized	Margarine, regular fat
Global	Uncategorized	Mayonnaise
Global	Uncategorized	Milk, condensed
Global	Uncategorized	Mineral water
Global	Uncategorized	Mixed Fruits in Syrup
Global	Uncategorized	Orange juice
Global	Uncategorized	Orange marmalade
Global	Uncategorized	Pineapple Jam
Global	Uncategorized	Potato chips
Global	Uncategorized	Sandwich biscuits/cookies
Global	Uncategorized	Strawberry/Apricot Jam
Global	Uncategorized	Thin Soya Sauce
Global	Uncategorized	Toffee
Global	Uncategorized	Tomato ketchup
Global	Uncategorized	Tomato paste (Large)
Global	Uncategorized	Tomato paste (Small)
Asia	Uncategorized	Black Pepper, powder
Asia	Uncategorized	Buffalo, without bones, non-specific cut
Asia	Uncategorized	Cake mix
Asia	Uncategorized	Carbonated Soft Drink, small bottle
Asia	Uncategorized	Chilies, dried
Asia	Uncategorized	Chinese cake/Moon cake
Asia	Uncategorized	Chocolate bar [Specified brand]

ICP product list	EAT-*Lancet* food group	ICP item name
Asia	Uncategorized	Coffee whitener [Specified brand 1], jar
Asia	Uncategorized	Coffee whitener [Specified brand 1], packet
Asia	Uncategorized	Cornflakes [Specified brand 1]
Asia	Uncategorized	Cornflakes [Specified brand 1], smaller package
Asia	Uncategorized	Cup cakes
Asia	Uncategorized	Dark soy sauce
Asia	Uncategorized	Decaffeinated coffee [Specified brand 1]
Asia	Uncategorized	Decaffeinated coffee, exclude [Specified brand 1]
Asia	Uncategorized	Doughnuts
Asia	Uncategorized	Fish sauce
Asia	Uncategorized	Fruit juice, not from concentrate, ready to drink
Asia	Uncategorized	Ghee, vegetable
Asia	Uncategorized	Infant powdered milk, box
Asia	Uncategorized	Infant powdered milk, tin
Asia	Uncategorized	Instant coffee, exclude [Specified brand 1]
Asia	Uncategorized	Jam, high fruit content
Asia	Uncategorized	Jam, low fruit content
Asia	Uncategorized	Malted beverage [Specified brand 1], glass Jar
Asia	Uncategorized	Malted beverage [Specified brand 1], glass Jar, small
Asia	Uncategorized	Malted beverage [Specified brand 1], packet
Asia	Uncategorized	Malted beverage [Specified brand 2 or 3], packet
Asia	Uncategorized	Malted beverage [Specified brand 2], tin
Asia	Uncategorized	Malted beverage [Specified brand 2], tin, small
Asia	Uncategorized	Malted beverage [Specified brand 3], glass jar
Asia	Uncategorized	Muffin
Asia	Uncategorized	Peanut butter
Asia	Uncategorized	Powdered Juice Mix [Specified brand]
Asia	Uncategorized	Savory pie
Asia	Uncategorized	Soft drinks, small bottle
Asia	Uncategorized	Sponge Cake
Asia	Uncategorized	Tamarind
Asia	Uncategorized	Tamarind, preserved
Asia	Uncategorized	Turmeric powder
Africa	Uncategorized	Baby food
Africa	Uncategorized	Bean Leaves
Africa	Uncategorized	Black Olives
Africa	Uncategorized	Black or white pepper corns
Africa	Uncategorized	Black or white pepper powder
Africa	Uncategorized	Blended coffee
Africa	Uncategorized	Bouillon/stock cube [Specified brand]
Africa	Uncategorized	Branded baby food, fruit based
Africa	Uncategorized	Carbonated Mineral Water
Africa	Uncategorized	Carbonated Soft Drink [Specified brands and model]

ICP product list	EAT-*Lancet* food group	ICP item name
Africa	Uncategorized	Carbonated Soft Drink, can
Africa	Uncategorized	Chilies
Africa	Uncategorized	Chocolate biscuit
Africa	Uncategorized	Chocolate croissant
Africa	Uncategorized	Cinnamon powder
Africa	Uncategorized	Cloves
Africa	Uncategorized	Cola Drink
Africa	Uncategorized	Condensed milk sweetened
Africa	Uncategorized	Coriander seeds
Africa	Uncategorized	Custard powder
Africa	Uncategorized	Dark chocolate
Africa	Uncategorized	Domestic Corn Based Baby Food
Africa	Uncategorized	Doughnuts
Africa	Uncategorized	Fresh Parsley
Africa	Uncategorized	Ginger juice (fresh)
Africa	Uncategorized	Ginger powder
Africa	Uncategorized	Gizzard
Africa	Uncategorized	Guava juice
Africa	Uncategorized	Ice cream cone
Africa	Uncategorized	Lemon-lime flavored Carbonated Soft Drink
Africa	Uncategorized	Lime juice
Africa	Uncategorized	Local Soft Drink
Africa	Uncategorized	Mango juice
Africa	Uncategorized	Milk chocolate
Africa	Uncategorized	Mustard Seed
Africa	Uncategorized	Orange Drink
Africa	Uncategorized	Orange juice - nectar
Africa	Uncategorized	Peppers
Africa	Uncategorized	Pineapple juice
Africa	Uncategorized	Pineapple juice freshly squeezed
Africa	Uncategorized	Red or green chili paste
Africa	Uncategorized	Regular chewing gum
Africa	Uncategorized	Rice Based Baby Food
Africa	Uncategorized	Sausage
Africa	Uncategorized	Sesame
Africa	Uncategorized	Shells
Africa	Uncategorized	Simple cookie
Africa	Uncategorized	Sponge Cake
Africa	Uncategorized	Tomato juice
Africa	Uncategorized	Tonic
Africa	Uncategorized	Wafers
Africa	Uncategorized	Wheat Based Baby Food
Africa	Uncategorized	White Vinegar

ICP product list	EAT-*Lancet* food group	ICP item name
LAC	Uncategorized	Chili sauce
LAC	Uncategorized	Cinnamon
LAC	Uncategorized	Cocoa Powder, bag
LAC	Uncategorized	Dulce de leche
LAC	Uncategorized	Fruit nectars (single flavor)
LAC	Uncategorized	Guava jam
LAC	Uncategorized	Guava jelly
LAC	Uncategorized	Hard candy, filled
LAC	Uncategorized	Instant coffee granulated
LAC	Uncategorized	Instant fruit-juice flavored drink, powder
LAC	Uncategorized	Mayonnaise in doypack
LAC	Uncategorized	Mustard
LAC	Uncategorized	Orange juice
LAC	Uncategorized	Sandwich biscuits/cookies packaged
LAC	Uncategorized	Sports drink
West-Asia	Uncategorized	Apple cider vinegar
West-Asia	Uncategorized	Baby milk, Powder (Nan standard)
West-Asia	Uncategorized	Baklava
West-Asia	Uncategorized	Biscuit Assortment
West-Asia	Uncategorized	Breakfast corn cereal
West-Asia	Uncategorized	Breakfast wheat cereal
West-Asia	Uncategorized	Caramels and toffees
West-Asia	Uncategorized	Cream biscuits
West-Asia	Uncategorized	Custard powder
West-Asia	Uncategorized	Domestic Garlic
West-Asia	Uncategorized	Doughnuts
West-Asia	Uncategorized	Dried Cardamom
West-Asia	Uncategorized	Dried Cinnamon
West-Asia	Uncategorized	Dried Coriander
West-Asia	Uncategorized	Dried Cumin
West-Asia	Uncategorized	Dried Ginger
West-Asia	Uncategorized	Fruit jellies
West-Asia	Uncategorized	Ground coffee
West-Asia	Uncategorized	Ice cream, Mixed
West-Asia	Uncategorized	Imported Garlic
West-Asia	Uncategorized	Instant Coffee
West-Asia	Uncategorized	Iodized salt
West-Asia	Uncategorized	Kaak (Bakssamat)
West-Asia	Uncategorized	Katayef
West-Asia	Uncategorized	Knefeh
West-Asia	Uncategorized	Liquid Tahini
West-Asia	Uncategorized	Mineral water, Carbonated water, 1 liter
West-Asia	Uncategorized	Mineral water, noncarbonated, 0.5liters

ICP product list	EAT-*Lancet* food group	ICP item name
West-Asia	Uncategorized	Orange juice (fresh)
West-Asia	Uncategorized	Oranges jam
West-Asia	Uncategorized	Peanut butter
West-Asia	Uncategorized	Regular cake(multiple) with cream topping
West-Asia	Uncategorized	Ripe(black) Olives
West-Asia	Uncategorized	Ripe(black) Olives, can or unpackaged
West-Asia	Uncategorized	Saffron
West-Asia	Uncategorized	Sanbousik
West-Asia	Uncategorized	Semi sweet baking chocolate
West-Asia	Uncategorized	Suckers lollipops
West-Asia	Uncategorized	Tahini
West-Asia	Uncategorized	Tonic water [Specified brand]
West-Asia	Uncategorized	Watermelon seeds
West-Asia	Uncategorized	White distilled vinegar

References

1. World Bank. Measuring the Real Size of the World Economy: The Framework, Methodology, and Results of the International Comparison Program—ICP. Washington, DC: World Bank; 2013.

2. UN. World Population Prospects: The 2017 Revision. Geneva: United Nations (UN), Department of Economic and Social Affairs, Population Division 2017.

3. Charrondiere UR, Stadlmayr B, Wijesinha-Bettoni R, Rittenschober D, Nowak V, Burlingame B. INFOODS contributions to fulfilling needs and meeting challenges concerning food composition databases. Procedia Food Science. 2013;2:35-45.

4. USDA. USDA National Nutrient Database for Standard Reference, Release 27 (slightly revised), Version Current: May 2015.2015.

5. Ahuja JK, Moshfegh AJ, Holden JM, Harris E. USDA food and nutrient databases provide the infrastructure for food and nutrition research, policy, and practice. The Journal of nutrition. 2012;143(2):241S-9S.

6. Padovani RM, Lima DM, Colugnati FA, Rodriguez-Amaya DB. Comparison of proximate, mineral and vitamin composition of common Brazilian and US foods. J Food Compost Anal. 2007;20(8):733-8.

7. Garcia V, Rona R, Chinn S. Effect of the choice of food composition table on nutrient estimates: a comparison between the British and American (Chilean) tables. Public Health Nutr. 2004;7(4):577-83.

8. Willett W, Rockström J, Loken B, Springmann M, Lang T, Vermeulen S, et al. Food in the Anthropocene: the EAT–Lancet Commission on healthy diets from sustainable food systems. The Lancet. 2019;Published Online January 16, 2019. http://dx.doi.org/10.1016/S0140-6736(18)31788-4:1-47.

9. FAO. FAO/INFOODS Global Food Composition Database for Fish and Shellfish Version 1.0 - uFiSh1.0. In: Nations FaAOotU, editor. Rome2016.

10. World Development Indicators (WDI) database. Data retrieved: 24 March 2019 [Internet]. 2019.

11. Deaton A. Measuring poverty in a growing world (or measuring growth in a poor world). Rev Econ Statist. 2005;87(1):1-19.

12. Beegle K, Christiaensen L, Dabalen A, Gaddis I. Poverty in a rising Africa: The World Bank; 2016.

13. Berkelaar M, Dirks J, Eikland K, Notebaert P, Ebert J, Gourvest H. lpSolve: Interface to Lp_solve v. 5.5 to solve linear/integer programs. R package version 5.6. 10. 2010.
